# Association between commensality practices and healthy food consumption in Primary Care: a cross-sectional study, Goiânia, 2022-2023

**DOI:** 10.1590/S2237-96222025v34e20240367.en

**Published:** 2025-10-27

**Authors:** Roberta Sena Reis, Raquel Machado Schincaglia, Mariana Martins Moreira, Jordanna de Souza Ferreira, Priscila Farage de Gouveia, Andréa Sugai Mortoza, Marilia Mendonça Guimarães, Maria do Rosário Gondim Peixoto, Luciana Bronzi de Souza

**Affiliations:** 1 Universidade Federal de Goiás, Faculdade de Nutrição, Goiânia, GO, Brazil

**Keywords:** Eating, Food Guides, Primary Health Care, Adult, Cross-Sectional Studies, Ingestión de Alimentos, Guías Alimentarias, Atención Primaria de Salud, Adulto, Estudios Transversales

## Abstract

**Objective:**

To verify association between adherence to the commensality practices recommended by the Dietary Guidelines for the Brazilian Population and healthy food consumption.

**Methods:**

This is a cross-sectional study with adults attending Primary Health Care in Goiânia. The healthy food consumption score (0-7 points) was reached by assigning 1 point for each “yes” answer for consumption of three healthy food markers and for each “no” answer for consumption of four unhealthy food markers. The recommended commensality practices score (0-3 points) was assessed by the sum of the habits of eating screen-free meals, eating at the table, and eating in the company of others. Sociodemographic and behavioral variables were assessed. Quantile regression was performed with a 5% significance level.

**Results:**

A total of 783 overweight adults, mostly mixed race women, were assessed. Beans were the healthy marker (74.8%), and sugar-sweetened drinks were the unhealthy marker (52.1%) most consumed the previous day. Eating with others (77.3%) was the most prevalent commensality practice. The median healthy food consumption score was 5 (4-6), and the median recommended commensality practice score was 2 (1-3). Among women, for each increase in one recommended commensality practice undertaken, the healthy food consumption score increased by 0.42 (95% confidence interval [95% CI] 0.18; 0.66) at the 10th percentile; by 0.31 (95% CI 0.13; 0.49) at the 25th percentile; by 0.17 (95%CI 0.04; 0.29) at the 50th percentile; and by 0.16 (95%CI 0.02-0.31) at the 75th percentile, but it was not associated with the 90th percentile. No associations were found among men.

**Conclusion:**

Commensality practices recommended by the Dietary Guidelines for the Brazilian Population were associated with healthier food consumption in the sample of adult women in Primary Care.

## Resumen

**Objetivo:**

Verificar la asociación entre la adherencia a las prácticas de comensalidad recomendadas por la Guía Alimentaria para la Población Brasileña y el consumo de alimentos saludables.

**Métodos:**

Estudio transversal con adultos que asistían a la Atención Primaria de Salud en Goiânia. La puntuación de consumo de alimentos saludables (0-7 puntos) se construyó asignando 1 punto por cada respuesta afirmativa al consumo de tres marcadores de alimentos saludables y por cada respuesta negativa al consumo de cuatro marcadores de alimentos no saludables. La puntuación de prácticas de comensalidad recomendadas (0-3 puntos) se evaluó mediante la suma de los hábitos de comer sin pantallas, comer en la mesa y comer en compañía. Se evaluaron variables sociodemográficas y conductuales. Se realizó una regresión cuantílica con un nivel de significancia del 5%.

**Resultados:**

Se evaluó a un total de 783 adultos con sobrepeso, en su mayoría mujeres de piel morena. Los frijoles fueron el marcador saludable (74,84%) y las bebidas azucaradas el marcador no saludable (52,11%) más consumidos el día anterior. Comer con otras personas (77,3%) fue la práctica de comensalidad más frecuente. La mediana de la puntuación de consumo de alimentos saludables fue de 5 (4-6), y la mediana de la puntuación de práctica de comensalidad recomendada fue de 2 (1-3). Entre las mujeres, por cada aumento en una práctica de comensalidad recomendada realizada, hubo un aumento en la puntuación de consumo de alimentos saludables de 0,42 (intervalo de confianza del 95% [IC del 95%] 0,18; 0,66) en el percentil 10, 0,31 (IC del 95% 0,13; 0,49) en el percentil 25; 0,17 (IC del 95% 0,04; 0,29) en el percentil 50; y 0,16 (IC del 95% 0,02-0,31) en el percentil 75, pero no se asoció con el percentil 90. No se encontraron asociaciones entre los hombres.

**Conclusión:**

Las prácticas de comensalidad recomendadas por la Guía Alimentaria para la Población Brasileña se asociaron con un consumo de alimentos más saludable en la muestra de mujeres adultas en Atención Primaria.

**Ethical aspects**

This research respected ethical principles, having obtained the following approval data:

Research Ethics Committee: Universidade Federal de Goiás

Opinion number: 5,213,102

Approval date: 26/1/2022

Certificate of Submission for Ethical Appraisal: 26118319.5.0000.5083

Informed Consent Form: Obtained from all participants prior to data collection.

## Introduction 

Eating practices are understood as the set of eating activities, which include meal planning, eating habits, and food consumption itself (1). These practices are related to individual aspects, such as the meanings, values, and symbolic representations attributed to food, as well as to the food environment (1,2).

The eating practices recommended by the Dietary Guidelines for the Brazilian Population (2014) include commensality (3) – originating from the Latin “mensa,” meaning “to share the table” – and a focus on how one eats, not just what one eats (4). Three commensality practices are presented: eating regularly and mindfully, eating in appropriate environments, and eating in company. Adopting these practices makes mealtimes pleasurable and communal.

The Guidelines address the context in which food is consumed and present dietary recommendations based on the Nova classification (not an acronym). This categorizes foods according to the extent and purpose of processing. Its recommendations address non-quantitative aspects of nutrition, focusing on foods and food groups and their impact on health (3). 

Despite the progress these dietary recommendations represent, an increase in the share of ultra-processed foods in total household calories has been observed in Brazil (5). This scenario highlights the need to monitor the relationship between eating practices and diet quality, as poor quality can increase the risk of chronic non-communicable diseases (6). Commensality practices are directly associated with consumption of unprocessed or minimally processed foods and inversely associated with consumption of processed and ultra-processed foods (7). In the context of Primary Health Care, studies evaluating commensality practices are necessary to identify potential and challenges within the catchment area for promoting adequate and healthy eating. This is where the greatest efforts to promote and protect healthy practices, including food intake, should be concentrated.

This study aimed to verify association between adherence to the commensality practices recommended by the Dietary Guidelines and healthy food consumption among adult Primary Health Care service users.

## Methods 

### Design 

This is a cross-sectional study related to the parent research project entitled “Food environment, physical activity, and associated factors in adults living in a large municipality.” This study aimed to investigate the existence of association between perceived food environment and physical activity, eating practices, nutritional status, and food security status among adults in Goiânia.

### Setting 

Goiânia is the capital city of the state of Goiás. According to the 2022 Demographic Census, the city has a geographic area of 729.296 km^2^ and 1,437,366 inhabitants. In 2010, the city had a high municipal human development index (0.799) (8). According to the e-Gestor website, average Primary Care coverage in Goiânia in 2022 was 51.0% and was organized into seven health districts (North, South, West, East, Southwest, Campinas-Center, and Northwest). In 2021, Goiânia had 22 basic health centers and 61 family health centers.

### Participants 

The parent study included users of the selected Primary Health Care centers, aged 18-59, of both sexes, who resided within the health district’s catchment area. Pregnant women and adults with amputations/orthopedic problems that prevented anthropometric measurements, psychiatric/neurological problems that prevented interviews, and those undergoing cancer treatment were excluded.

### Sample size

The sample size calculation was performed on the OpenEpi platform, considering 50.0% prevalence for the outcomes of the parent study, 5.0% absolute error, a 95.0% confidence level, a design effect of 1.5, and an estimated population of 876,794 adults (based on the 2022 Demographic Census and the projection of 61.0% of adults in Goiânia) (9). This resulted in a minimum sample of 576 individuals, which was increased by 20.0%, so that the final required sample was 691 individuals.

Data collection for the parent survey was completed within a year, with 968 individuals interviewed, distributed proportionally to the number of inhabitants in the health districts. Two individuals were excluded due to questionnaires left blank, as were 183 who did not answer the questions related to commensality practices and/or food consumption markers, resulting in a total of 783 individuals. The 183 excluded individuals were compared with the 783 included individuals, revealing a significant difference in the proportion of males: 25.68% among the excluded individuals and 18.39% among the included individuals (p-value 0.030). No differences were identified for age, race/skin color, schooling, monthly family income, nutritional status, tobacco use, alcohol use or physical activity.

### Data collection

Participants were randomly selected in the waiting room while awaiting treatment at the Primary Health Care centers.

Data collection was conducted in person between May 2022 and May 2023 at 13 facilities (one basic health center and 12 family health centers) distributed across the seven health districts. In each district, two health centers with the highest number of appointments were selected. In the South District, there were two possible health centers for selection, but one of them declined participation.

The data collection schedule was defined based on the seven health districts, so as to cover one district per week. A draw was made every seven weeks to determine the order in which these districts would be visited. After the end of a seven-week cycle, a new draw was made to ensure randomness and seasonality in data collection. The cycles were repeated until the expected sample size for each district was obtained.

Data collection was performed by administering a structured and standardized questionnaire. A pilot study was conducted to test methods and instruments, and its respondents were included in the study sample.

### Variables 

The study outcome was the food consumption markers score, built based on the Food and Nutrition Surveillance System instrument (10). This instrument considered the previous day’s consumption of three healthy food groups—beans; fresh fruit (except fruit juice); and vegetables and greens (except potatoes, cassava, taro and yam)—and four unhealthy food groups—hamburgers and/or charcuterie (ham, mortadella, salami, sausage, hot dog sausage); sugar-sweetened drinks (soda, carton juice, powdered juice, carton coconut water, guarana/redcurrant syrups, fruit juice with added sugar); instant noodles, packaged snacks or savory biscuits (crackers); and stuffed cookies, sweets/treats (candies, lollipops, gum, caramel, gelatin). Respondents could answer yes or no for each group.

In the scoring assessment, 1 point was assigned for each “yes” answer regarding consumption of each of the three healthy food groups and for each “no” answer regarding consumption of each of the four unhealthy food groups. Each participant’s score ranged from 0 to 7 points, with a higher score indicating a healthier food intake. The food consumption markers score used was based on Louzada et al. (11), who evaluated healthy (0 to 3) and unhealthy (0 to 4) consumption markers from a scoring perspective, and this showed good potential to reflect overall diet quality. Our study used the same perspective, although with a combined response category for food consumption markers (ranging from 0 to 7).

The exposure variables were the commensality practices recommended by the Dietary Guidelines: having the habit of screen-free meals, including TV, computer, and/or cell phone (yes, no); having the habit of eating at the table (yes, no); and having the habit of eating with family or people living in the household (yes, no). For each “yes” reported, 1 point was assigned. The points were added together, and each participant’s score ranged from 0 to 3. The higher the score, the greater the number of recommended commensality practices undertaken by the respondent.

The independent variables were: age (complete years); sex (male, female); race/skin color (White, Black, mixed race, Asian); schooling level (up to elementary school, high school, and higher education); monthly family income, expressed according to the minimum wage at the time (<1, 1, 2-3, 4-5, >5); body mass index (underweight <18.5; healthy weight 18.5-24.9; overweight 25.0-29.9; obese ≥30.0) (12); tobacco use (yes, no); alcohol use (yes, no); and perceived level of physical activity (sedentary, moderate, active, very active).

The body mass index was calculated by dividing weight (kilograms) by height (meters) squared (12). Weight and height were either measured or self-reported (based on the last time the respondents weighed themselves), depending on whether equipment was available on the day the data was collected.

### Statistical analysis

The database was built using Epi Info, version 7.0, with double data entry. Data analysis was performed using Stata, version 12.0, and the data were presented as absolute (n) and relative (%) frequencies, or median and interquartile range (p25-p75). Given the discrete or non-normal characteristics of the numerical variables, the Mann-Whitney U or Kruskal-Wallis test was used for comparison between groups. Pearson’s chi-square or Fisher’s exact tests were used to compare proportions between groups. Spearman’s correlation (ρ) was calculated. 

Crude and adjusted quantile regression was applied and adjusted for the total sample and stratified by sex, using the food consumption score as the outcome, the recommended commensality practices as exposure, and the following variables for adjustment: age, sex (only for the total sample), schooling, body mass index, perceived physical activity level, alcohol use and tobacco use. The regression analysis was performed using the 10th, 25th, 50th, 75th and 90th percentiles of the healthy food consumption score. This analysis was chosen because it allowed us to assess changes in distribution at different percentiles when the outcome was not normal. The unstandardized effect size (t) was calculated by the ratio of the coefficient (α) to the standard error (SE) of the adjusted quantile regression. We adopted a 5% significance level.

## Results 

Of the 783 participants, the majority were aged between 36 and 59 years (n=444; 57.0%), were female (n=639; 81.6%) and self-reported mixed race (n=468; 59.9%), had completed high school (n=401; 51.3%), had a monthly family income of up to 3 minimum wages (n=616; 82.5%), were overweight (n=446; 58.6%), did not smoke tobacco (n=666; 89.2%), did not consume alcohol (n=460; 61.6%) and had a self-perceived level of physical activity classified as sedentary (n=398; 52.6%) (Table 1).

**Table 1:** Sociodemographic and behavioral characteristics according to healthy food consumption markers of adult Primary Health Care service users. Goiânia, 2022-2023 (n=783)

Variables	**Total sample** (n=783)	Healthy markers								
		Beans (n=783)			Fresh fruit (n=783)			Vegetables and greens (n=783)		
	n (%)	Yes n (%)	No n (%)	p-value	Yes n (%)	No n (%)	p-value	Yes n (%)	No n (%)	p-value
**Total sample^f^**	-	586 (74.8)	197 (25.2)	-	468 (59.8)	315 (40.2)	-	545 (69.6)	238 (30.4)	-
**Age (years)^a^** (n=779)				0.146^c^			0.001^c^			0.002^c^
18-35	335 (43.0)	242 (41.5)	93 (47.5)		177 (38.1)	158 (50.3)		213 (39.3)	122 (51.5)	
36-59	444 (57.0)	341 (58.5)	103 (52.5)		288 (61.9)	156 (49.7)		329 (60.7)	115 (48.5)	
**Sex** (n=783)				0.961^c^			0.354^c^			0.450^c^
Female	639 (81.6)	478 (81.6)	161 (81.7)		377 (80.6)	262 (83.2)		441 (80.9)	198 (83.2)	
Male	144 (18.4)	108 (18.4)	36 (18.3)		91 (19.4)	53 (16.8)		104 (19.1)	40 (16.8)	
**Race/skin color** (n=781)				0.033^d^			0.002^c^			0.514^c^
White	167 (21.4)	114 (19.5)	53 (26.9)		101 (21.6)	66 (21.0)		118 (21.7)	49 (20.6)	
Black	111 (14.2)	80 (13.7)	31 (15.8)		66 (14.1)	45 (14.3)		72 (13.3)	39 (16.4)	
Mixed race	468 (59.9)	359 (61.5)	109 (55.3)		290 (62.1)	178 (56.7)		331 (61.0)	137 (57.6)	
Asian	35 (4.5)	31 (5.3)	4 (2.0)		10 (2.2)	25 (8.0)		22 (4.0)	13 (5.4)	
**Schooling** (n=781)				0.076^c^			0.017^c^			0.062^c^
Up to complete elementary	264 (33.8)	200 (34.2)	64 (32.5)		149 (31.9)	115 (36.6)		173(31.8)	91 (38.2)	
Complete high school	401 (51.3)	307 (52.6)	94 (47.7)		235 (50.3)	166 (52.9)		280(51.6)	121 (50.9)	
Complete higher education	116 (14.9)	77 (13.2)	39 (19.8)		83 (17.8)	33 (10.5)		90(16.6)	26 (10.9)	
**Monthly family income (minimum wages)** (n=747)				0.610^c^			0.021^c^			0.007^c^
<1	55 (7.4)	42 (7.5)	13 (6.9)		31 (6.9)	24 (8.0)		32 (6.1)	23 (10.1)	
1	227 (30.4)	175 (31.4)	52 (27.4)		120 (26.8)	107 (35.7)		146 (28.1)	81 (35.7)	
2-3	334 (44.7)	249 (44.7)	85 (44.7)		207 (46.3)	127 (42.3)		237 (45.6)	97 (42.7)	
4-5	77 (10.3)	53 (9.5)	24 (12.6)		48 (10.7)	29 (9.7)		60 (11.5)	17 (7.5)	
>5	54 (7.2)	38 (6.8)	16 (8.4)		41 (9.2)	13 (4.3)		45 (8.6)	9 (4.0)	
**Body mass index** (kg/m^2^)	26.2 (23.1-30.4)^a^	27.0 (5.8)	27.8 (6.0)	0.094^b^	27.2 (5.8)	27.2 (6.0)	0.956^b^	27.4 (6.0)	26.6 (5.7)	0.093^b^
**Nutritional status** (n=761)				0.214^d^			0.848^c^			0.839^c^
Underweight	28 (3.7)	25 (4.4)	3 (1.6)		16 (3.5)	12 (4.0)		20 (3.8)	8 (3.5)	
Healthy weight	287 (37.7)	221 (38.6)	66 (35.1)		176 (38.5)	111 (36.5)		194 (36.7)	93 (40.1)	
Overweight	238 (31.3)	176 (30.7)	62 (33.0)		138 (30.2)	100 (32.9)		169 (31.9)	69 (29.7)	
Obese	208 (27.3)	151 (26.3)	57 (30.3)		127 (27.8)	81 (26.6)		146 (27.6)	62 (26.7)	
**Tobacco use** (n=747)				0.868^c^			<0.001^c^			0.032^c^
Yes	81 (10.8)	60 (10.7)	21 (11.2)		31 (6.9)	50 (16.7)		48 (9.2)	33 (14.5)	
No	666 (89.2)	499 (89.3)	167 (88.8)		417 (93.1)	249 (83.3)		472 (90.8)	194 (85.5)	
**Alcohol use** (n=747)				0.078^c^			<0.001^c^			0.770^c^
Yes	287 (38.4)	205 (36.6)	82 (43.9)		145 (32.4)	142 (47.5)		198 (38.1)	89 (39.2)	
No	460 (61.6)	355 (63.4)	105 (56.1)		303 (67.6)	157 (52.5)		322 (61.9)	138 (60.8)	
**Physical activity** (n=757)				0.637^d^			<0.001^d^			0.004^d^
Sedentary	398 (52.6)	299 (52.4)	99 (52.9)		202 (44.8)	196 (64.1)		256 (48.6)	142 (61.7)	
Moderate	182 (24.0)	140 (24.6)	42 (22.5)		122 (27.0)	60 (19.6)		131 (24.9)	51 (22.2)	
Active	157 (20.7)	114 (20.0)	43 (23.0)		111 (24.6)	46 (15.0)		124 (23.5)	33 (14.4)	
Very active	20 (2.6)	17 (3.0)	3 (1.6)		16 (3.6)	4 (1.3)		16 (3.0)	4 (1.7)	

^a^Values presented as medians (interquartile range); ^b^Mann-Whitney U test; ^c^Pearson’s chi-square test; ^d^Fisher’s exact test; ^e^Kruskal-Wallis test; ^f^Some variables had n<783 due to missing data.

Regarding healthy food consumption markers, 74.8% (n=586) of participants reported having consumed beans the previous day; 59.8% (n=468) fresh fruit; and 69.6% (n=545) vegetables and greens (Table 1). Regarding unhealthy food consumption markers, sugar-sweetened drinks were the most frequently consumed marker the previous day (n=408; 52.1%). There was a lower frequency of consumption of stuffed cookies, sweets or treats (n=224; 28.6%), hamburger and/or charcuterie (n=152; 19.4%), and instant noodles, packaged snacks, or savory biscuits (n=143; 18.3%) (Table 2). The differences in the food consumption markers, according to sociodemographic and behavioral variables, are shown in Tables 1 and 2. The healthy food consumption score had a median of 5 (4-6) points in the total sample and differed according to age (p-value<0.001), sex (p-value 0.032), tobacco use (p-value 0.005), alcohol use (p-value<0.001) and level of physical activity (p-value 0.002) (Table 2).

**Table 2:** Sociodemographic and behavioral characteristics according to unhealthy food consumption markers of Primary Health Care service users. Goiânia, 2022-2023 (n=783)

Variables	Unhealthy markers												Healthy food consumption score (n=783)	
	Hamburger and/or charcuterie (n=783)			Sugar-sweetened drinks (n=783)			Instant noodles, packaged snacks or savory biscuits (n=783)			Stuffed cookies, sweets or treats (n=783)				
	Yes n (%)	No n (%)	p-value	Yes n (%)	No n (%)	p-value	Yes n (%)	No n (%)	p-value	Yes n (%)	No n (%)	p-value	Median (IQR)	p-value
**Total sample^f^**	152 (19.4)	631 (80.6)	-	408 (52.1)	375 (47.9)	-	143 (18.3)	640 (81.7)	-	224 (28.6)	559 (71.4)	-	5 (4-6)	-
**Age (years)^a^ (n=779)**			0.013^c^			<0.001^c^			<0.001^c^			<0.001^c^		<0.001^b^
18-35	79 (52.0)	256 (40.8)		210 (51.7)	125 (33.5)		82 (57.3)	253 (39.8)		128 (57.7)	207 (37.2)		4 (3-6)	
36-59	73 (48.0)	371 (59.2)		196 (48.3)	248 (66.5)		61 (42.7)	383 (60.2)		94 (42.3)	350 (62.8)		5 (4-6)	
**Sex** (n=783)			0.005^c^			0.002^c^			0.173^c^			0.016^c^		0.032^b^
Female	112 (73.7)	527 (83.5)		316 (77.4)	323 (86.1)		111 (77.6)	528 (82.5)		171 (76.3)	468 (83.7)		5 (4-6)	
Male	40 (26.3)	104 (16.5)		92 (22.6)	52 (13.9)		32 (22.4)	112 (17.5)		53 (23.7)	91 (16.3)		5 (3.5-6)	
**Race/skin color** (n=781)			0.188^d^			0.276^c^			0.802^d^			0.978^c^		0.631^e^
White	25 (16.4)	142 (22.6)		95 (23.3)	72 (19.3)		31 (21.7)	136 (21.3)		47 (21.1)	120 (21.5)		5 (4-6)	
Black	25 (16.4)	86 (13.7)		63 (15.5)	48 (12.8)		21 (14.7)	90 (14.1)		30 (13.4)	81 (14.5)		5 (4-6)	
Mixed race	98 (64.5)	370 (58.8)		232 (57)	236 (63.1)		87 (60.8)	381 (59.7)		136 (61.0)	332 (59.5)		5 (4-6)	
Asian	4 (2.6)	31 (4.9)		17 (4.2)	18 (4.8)		4 (2.8)	31 (4.9)		10 (4.5)	25 (4.5)		5 (4-6)	
**Schooling** (n=781)			0.495^c^			0.782^c^			0.393^c^			0.087^c^		0.247^e^
Up to complete elementary	46 (30.3)	218 (34.7)		133 (32.7)	131 (35.0)		50 (35.0)	214 (33.5)		70 (31.2)	194 (34.8)		5 (4-6)	
Complete high school	80 (52.6)	321 (51.0)		213 (52.3)	188 (50.3)		77 (53.8)	324 (50.8)		128 (57.1)	273 (49.0)		5 (4-6)	
Complete higher education	26 (17.1)	90 (14.3)		61 (15.0)	55 (14.7)		16 (11.2)	100 (15.7)		26 (11.6)	90 (16.2)		5 (4-6)	
**Monthly family income (minimum wages)** (n=747)			0.076^c^			0.212^c^			0.257^d^			0.787c		0.142^e^
<1	9 (6.2)	46 (7.7)		25 (6.5)	30 (8.3)		9 (6.9)	46 (7.5)		13 (6.1)	42 (7.9)		5 (4-6)	
1	38 (26.0)	189 (31.4)		105 (27.2)	122 (33.8)		44 (33.6)	183 (29.7)		64 (29.9)	163 (30.6)		5 (4-6)	
2-3	69 (47.3)	265 (44.1)		183 (47.4)	151 (41.8)		58 (44.3)	276 (44.8)		95 (44.4)	239 (44.8)		5 (4-6)	
4-5	23 (15.7)	54 (9.0)		44 (11.4)	33 (9.2)		16 (12.2)	61 (9.9)		26 (12.1)	51 (9.6)		5 (4-6)	
>5	7 (4.8)	47 (7.8)		29 (7.5)	25 (6.9)		4 (3.0)	50 (8.1)		16 (7.5)	38 (7.1)		5.5 (4-6)	
**Body mass index** (kg/m^2^)	27.6 (6.0)	27.1 (5.9)	0.404^c^	27.0 (5.9)	27.3 (5.8)	0.465^b^	26.2 (6.2)	27.4(5.8)	0.007^b^	26.9 (6.5)	27.3 (5.6)	0.126^b^		-
**Nutritional status** (n=761)			0.359^c^			0.812^c^			0.120^c^			0.184^c^		0.709^e^
Underweight	6 (4.0)	22(3.6)		17 (4.2)	11 (3.0)		9 (6.5)	19 (3.1)		11 (5.0)	17 (3.1)		4 (4-6)	
Healthy weight	56 (37.6)	231 (37.8)		148 (37.0)	139 (38.5)		58 (41.7)	229 (36.8)		92 (42.0)	195 (36.0)		5 (4-6)	
Overweight	39 (26.2)	199 (32.5)		127 (31.8)	111 (30.8)		40 (28.8)	198 (31.8)		60 (27.4)	178 (32.9)		5 (4-6)	
Obese	48 (32.2)	160 (26.1)		108 (27.0)	100 (27.7)		32 (23.0)	176 (28.3)		56 (25.6)	152 (28.0)		5 (4-6)	
**Tobacco use** (n=747)			0.144^c^			0.952^c^			0.702^c^			0.599^c^		0.005^b^
Yes	21 (14.2)	60 (10.0)		42 (10.9)	39 (10.8)		16 (11.8)	65 (10.6)		25 (11.8)	56 (10.5)		4 (4-6)	
No	127 (85.8)	539 (90.0)		343 (89.1)	323 (89.2)		120 (88.2)	546 (89.4)		187 (88.2)	479 (89.5)		5 (4-6)	
**Alcohol use** (n=747)			0.002^c^			0.013^c^			0.884^c^			0.097^c^		<0.001^b^
Yes	73 (49.7)	214 (35.7)		164 (42.7)	123 (33.9)		53 (39.0)	234 (38.3)		91 (43.1)	196 (36.6)		5 (4-6)	
No	74 (50.3)	386 (64.3)		220 (57.3)	240 (66.1)		83 (61.0)	377 (61.7)		120 (56.9)	340 (63.4)		5 (4-6)	
**Physical activity** (n=757)			0.979^d^			0.175^c^			0.052^d^			0.235^c^		0.002^e^
Sedentary	76 (51.4)	322 (52.9)		220 (56.0)	178 (48.9)		76 (54.3)	322 (52.2)		111 (51.9)	287 (52.9)		5 (4-6)	
Moderate	36 (24.3)	146 (24.0)		91 (23.1)	91 (25.0)		24 (17.1)	158 (25.6)		58 (27.1)	124 (22.8)		5 (4-6)	
Active	32 (21.6)	125 (20.5)		71 (18.1)	86 (23.6)		38 (27.1)	119 (19.3)		37 (17.3)	120 (22.1)		5 (4-6)	
Very active	4 (2.7)	16 (2.6)		11 (2.8)	9 (2.5)		2 (1.4)	18 (2.9)		8 (3.7)	12 (2.2)		5 (4-6.5)	

^a^Values presented as medians (interquartile range); ^b^Mann-Whitney U test; ^c^Pearson’s chi-square test; ^d^Fisher’s exact test; ^e^Kruskal-Wallis test; ^f^Some variables had n<783 due to missing data.

**Table 3:** Commensality practices according to healthy food consumption markers of adult Primary Health Care service users. Goiânia, 2022-2023 (n=783)

Variables	Total sample (n=783)	Healthy markers								
		Beans (n=783)			Fresh fruit (n=783)			Vegetables and greens (n=783)		
	n (%)	Yes n (%)	No n (%)	p-value	Yes n (%)	No n (%)	p-value	Yes n (%)	No n (%)	p-value
**Total sample^f^**	-	586 (74.8)	197 (25.2)	-	468 (59.8)	315 (40.2)	-	545 (69.6)	238 (30.4)	-
**Habit of having screen-free meals**				0.016^c^			<0.001^c^			0.001^c^
Yes	476 (60.8)	342 (58.4)	134 (68.0)		261 (55.8)	215 (68.2)		311 (57.1)	165 (69.3)	
No	307 (39.2)	244 (41.6)	63 (32.0)		207 (44.2)	100 (31.8)		234 (42.9)	73 (30.7)	
**Habit of having eating food at the table**				0.527^c^			<0.001^c^			0.001^c^
Yes	490 (62.6)	363 (61.9)	127 (64.5)		327 (69.9)	163 (51.8)		361 (66.2)	129 (54.2)	
No	293 (37.4)	223 (38.1)	70 (35.5)		141 (30.1)	152 (48.2)		184 (33.8)	109 (45.8)	
**Habit of having eating food with company**				0.222^c^			0.145^c^			0.003^c^
Yes	605 (77.3)	459 (78.3)	146 (74.1)		370 (79.1)	235 (74.6)		437 (80.2)	168 (70.6)	
No	178 (22.7)	127 (21.7)	51 (25.9)		98 (20.9)	80 (25.4)		108 (19.8)	70 (29.4)	
**Score for recommended commensality practices** (n=783) Median (IQR)	2 (1-3)	2 (1-3)	2 (1-2)	0.156^b^	2 (1-3)	2 (1-2)	<0.001^b^	2 (1-3)	2 (1-2)	<0.001^b^

^a^Values presented as medians (interquartile range); ^b^Mann-Whitney U test; ^c^Pearson’s chi-square test; ^d^Fisher’s exact test; ^e^Kruskal-Wallis test; ^f^Some variables had n<783 due to missing data.

Differences in the prevalence of food consumption markers were observed according to commensality practices (Tables 3 and 4). The median score for recommended commensality practices in the total sample was 2 (1-3) points; 39.2% (n=307) of adults reported the habit of eating screen-free meals; 62.6% (n=490) the habit of eating at the table; and 77.3% (n=605) eating with company. The healthy food consumption score differed according to the habit of eating screen-free meals (p-value<0.001) and the habit of eating at the table (p-value<0.001). The habit of eating with company was not related to the healthy food consumption score (p-value 0.199) (Table 4).

**Table 4:** Commensality practices according to unhealthy food consumption markers of adult Primary Health Care service users. Goiânia, 2022-2023 (n=783)

Variables	Unhealthy markers												Healthy food consumption score (n=783)	
	Hamburger and/or charcuterie (n=783)			Sugar-sweetened drinks (n=783)			Instant noodles, packaged snacks or savory biscuits (n=783)			Stuffed cookies, sweets or treats (n=783)				
	Yes n (%)	No n (%)	p-value	Yes n (%)	No n (%)	p-value	Yes n (%)	No n (%)	p-value	Yes n (%)	No n (%)	p-value	Median (IQR)	p-value
**Total sample^f^**	152 (19.4)	631 (80.6)	-	408 (52.1)	375 (47.9)	-	143 (18.3)	640 (81.7)	-	224 (28.6)	559 (71.4)	-	5 (4-6)	-
**Habit of having screen-free meals**			0.001^c^			<0.001^c^			0.056^c^			0.001^c^		<0.001^b^
Yes	110 (72.4)	366 (58.0)		272 (66.7)	204 (54.4)		97 (67.8)	379 (59.2)		156 (69.6)	320 (57.2)		5 (5-6)	
No	42 (27.6)	265 (42.0)		136 (33.3)	171(45.6)		46 (32.2)	261 (40.8)		68 (30.4)	239 (42.8)		5 (4-6)	
**Habit of having eating food at the table**			0.591^c^			0.010^c^			0.922^c^			0.722^c^		<0.001^b^
Yes	98 (64.5)	392 (62.1)		238 (58.3)	252 (67.2)		90 (62.9)	400 (62.5)		138 (61.6)	352 (63.0)		5 (4-6)	
No	54 (35.5)	239 (37.9)		170 (41.7)	123 (32.8)		53 (37.1)	240 (37.5)		86 (38.4)	207 (37.0)		5 (4-6)	
**Habit of having eating food with company**			0.923^c^			0.701^c^			0.320^c^			0.264^c^		0.199^b^
Yes	117 (77.0)	488 (77.3)		313 (76.7)	292 (77.9)		115 (80.4)	490 (76.6)		179 (79.9)	426 (76.2)		5 (4-6)	
No	35 (23.0)	143 (22.7)		95 (23.3)	83 (22.1)		28 (19.6)	150 (23.4)		45 (20.1)	133 (23.8)		5 (4-6)	
**Score for recommended commensality practices** (n=783) Median (IQR)	2 (1-2)	2 (1-3)	0.149^b^	2 (1-2)	2 (1-3)	0.001^b^	2 (1-2)	2 (1-3)	0.526^b^	2 (1-2)	2 (1-3)	0.163^b^	-	-

^a^Values presented as medians (interquartile range); ^b^Mann-Whitney U test; cPearson’s chi-square test; ^d^Fisher’s exact test; ^e^Kruskal-Wallis test; fSome variables had n<783 due to missing data.

The score for recommended commensality practices differed according to age (p-value<0.001), race/skin color (p-value 0.003), tobacco use (p-value <0.001), and physical activity level (p-value <0.001). Screen-free food consumption was higher among older participants (p-value <0.001), those who did not consume alcohol (p-value 0.035), and those who were sedentary (p-value 0.005). Eating at a table was more frequent among older participants (p-value 0.006), those of mixed race/skin color (p-value <0.001), those with an income between 2 and 3 minimum wages (p-value 0.001), those who did not use tobacco (p-value <0.001), and those who were sedentary (p-value 0.000). Eating in company was greater among those who did not use tobacco (p-value 0.001) (Supplementary Table 1).

There was positive correlation between the recommended commensality practices scores and the healthy food consumption scores (ρ=0.208, p-value<0.001) (data not shown).

In the analysis adjusted for the total sample, for each additional recommended commensality practice, the healthy food consumption score increased by 0.33 (95%CI 0.11; 0.56, p-value 0.004, t=2.91) at the 10th percentile; by 0.30 (95%CI 0.14; 0.47, p-value<0.001, t=3.65) at the 25th percentile; by 0.17 (95%CI 0.06; 0.29, p-value=0.004, t=2.91) at the 50th percentile; and by 0.15 (95%CI 0.02; 0.28, p-value 0.019, t=2.34) at the 75th percentile, however it was not associated (95%CI 0.13; -0.08; 0.34, p-value 0.222, t=1.22) with the 90th percentile of the healthy food consumption score distribution. The adjusted analysis stratified by sex showed that, for females, for each additional recommended commensality practice, the healthy food consumption score increased by 0.42 (95%CI 0.18; 0.66, p-value 0.001, t=3.46) at the 10th percentile; by 0.31 (95%CI 0.13; 0.49, p-value 0.001, t=3.44) at the 25th percentile; by 0.17 (95%CI 0.04; 0.29, p-value 0.008, t=2.66) at the 50th percentile; and by 0.16 (95%CI 0.02-0.31, p-value 0.027, t=2.21) at the 75th percentile, but it was not associated (0.17; 95%CI -0.04; 0.38, p-value 0.123, t=1.55) with the 90th percentile of the healthy food consumption score distribution. No associations were found for male sex (Table 5).

**Table 5:** Coefficient with 95% confidence intervals (95%CI) of association between commensality practices and distribution percentiles of previous day food consumption markers of adult Primary Health Care service users. Goiânia, 2022-2023

Model	10^th^ percentile		25^th^ percentile		50^th^ percentile		75^th^ percentile		90^th^ percentile	
	Coefficient (95%CI)	p-value	Coefficient (95%CI)	p-value	Coefficient (95%CI)	p-value	Coefficient (95%CI)	p-value	Coefficient (95%CI)	p-value
Total										
Crude model^a^ (n=783)	0 (-0.23; 0.23)	1.000	0.50 (0.29; 0.71)	<0.001	0 (-0.14; 0.14)	1.000	0 (-0.09; 0.09)	1.000	0 (-0.12; 0.12)	1.000
Adjusted model^b^ (n=710)	0.33 (0.11; 0.56)	0.004	0.30 (0.14; 0.47)	<0.001	0.17 (0.06; 0.29)	0.004	0.15 (0.02; 0.28)	0.019	0.13 (-0.08; 0.34)	0.222
**Sex female**										
Crude model^a^ (n=639)	0.5 (0.26; 0.74)	<0.001	0.50 (0.28; 0.72)	<0.001	0 (-0.18; 0.18)	1.000	0 (-0.09; 0.09)	1.000	0 (-0.12; 0.12)	1.00
Adjusted model^b^ (n-576)	0.42 (0.18; 0.66)	0.001	0.31 (0.13; 0.49)	0.001	0.17 (0.04; 0.29)	0.008	0.16 (0.02; 0.31)	0.027	0.17 (-0.04; 0.38)	0.123
**Sex male**										
Crude model^a^ (n=144)	0 (-0.54; 0.54)	1.000	0.33 (-0.06; 0.73)	0.101	0.33 (-0.03; 0.70)	0.075	0 (-0.29; 0.29)	1.000	0.33 (-0.32-0.99)	0.318
Adjusted model^b^ (n=134)	-0.02 (-0.54; 0.50)	0.931	0.42 (-0.06; 0.90)	0.089	0.14 (-0.25; 0.52)	0.481	0.04 (-0.37; 0.45)	0.834	-0.15 (-0.45; 0.14)	0.311

^a^Quantile regression analysis taking the healthy food score (0-7 points) as the outcome and the recommended commensality practices score (0-3 points) as exposure in crude models; ^b^Quantile regression analysis taking the healthy food score (0-7 points) as the outcome and the recommended commensality practices score (0-3 points) as exposure in models adjusted for age, sex (only for the total sample), schooling, body mass index, perceived physical activity level, alcohol use and tobacco use.

## Discussion 

This study found that adherence to commensality practices recommended by the Dietary Guidelines, such as eating at the table, in company, and without screen time, was associated with healthier food consumption in women. Adjusted analyses showed that with each additional practice, the healthy consumption score increased, except among those whose scores were already high. This association was not observed in men.

In this study, a score was established to measure commensality practices, with a moderate median. Eating with company was the most frequent practice, followed by eating at a table, while screen-free meals were the least prevalent. The median healthy food consumption score was high due to the higher frequency of consumption of unprocessed, minimally processed foods and culinary preparations such as foods from the group that includes fresh fruit, vegetables, greens, and beans, in accordance with the golden rule of the Dietary Guidelines (3).

Although national data reveal that bean consumption has declined over the years (5), this was the most prevalent health marker in this study. Bean consumption is an important habit for diet quality, as it favors the consumption of healthy foods such as rice, salad, and meat (13). However, it was found that more than half of the sample consumed sugar-sweetened beverages, such as soft drinks, artificial fruit juices, and sweetened fruit juices. Fruit juice consumption is common during meals (14). However, the Dietary Guidelines recommends the consumption of fruit itself rather than fruit juices, as the latter have less fiber and provide less satiety (3). Despite the cumulative reduction of 43.8% from 2007 to 2019 in the consumption of soft drinks/artificial juices, this remains high (15), as can be seen.

Individuals over 36 years of age, female, highly active, who do not use tobacco or alcohol, who eat without looking at screens, and who consume meals at the table had higher healthy eating scores. A study found a trend toward improved eating habits among Brazilian adults with increasing age in both sexes (16). In the National Health Survey (2019), according to sex, age group, and education level, women, younger individuals, and those with higher education levels had a higher proportion of the recommended intake of fruit, vegetables, greens and fish, and a lower proportion of soft drinks, artificial juices, and sweets (17). Males were associated with higher consumption of ultra-processed foods. These factors should be considered in public policies that promote dietary equity (18).

Quantile regression analyses adjusted for sociodemographic, lifestyle and nutritional status variables were used to investigate association between the recommended commensality practices score and the healthy food consumption score at different points in the distribution. This approach proved appropriate given the asymmetric distribution of the healthy food consumption score.

In the models adjusted for the total sample, it was found that, with each additional recommended commensality practice, there was a significant increase in the healthy food consumption score at the 10th, 25th, 50th and 75th percentiles of the distribution, while no association was detected at the 90th percentile. These results showed that individuals in the lowest percentiles of distribution benefited most from adopting commensality practices, possibly due to the greater room for improvement in their food consumption. For individuals with healthier diets, the possibility of additional dietary changes was more limited (19). Other factors may protect these individuals, such as food and nutrition education, dietary self-control, health concerns, availability and access to healthy foods, healthy lifestyles, mental and emotional health (20-22), eating mindfully, eating regularly, and dedicating more time to eating (3).

The analysis stratified by sex revealed distinct results. Among women, there was positive association between the commensality practice score and the healthy food score at several percentiles, except at the 90th percentile of the distribution, as observed in the model for the total sample. No significant association was observed among men, even after adjusting for potential confounders. The lack of association among men may be related to the smaller male sample size, lower adherence to commensality practices, or other unadjusted factors.

Differences between the sexes may include behavioral, social and cultural aspects. Women are more involved in commensality and activities involving food, which favors greater culinary skills and influences healthier food consumption. Male food consumption may be more influenced by factors such as convenience, satiety, and automatic habits, which can mitigate or neutralize the impact of commensality in this group (23). Challenging this cultural pattern can encourage male participation in these practices and promote healthy food consumption among men (24).

When exposed to health guidance, women tend to adopt healthier behaviors (25). Eating while watching television is a practice that contrasts with sharing meals at the table and in company. Eating in company improves dietary quality, strengthens social and cultural bonds, promotes a healthier lifestyle (26,27), and is associated with lower risk of obesity (28). From a bioarchaeological perspective, sharing food and participating in communal meals constitute acts that connect human beings as biological organisms to their social nature (29), in addition to the greater sense of pleasure provided by eating (30). Adhering to the commensality practices recommended by the Dietary Guidelines has been associated with better dietary quality (7).

The results of this study stressed the importance of Primary Health Care teams promoting commensality practices as a strategy to improve dietary quality and align it with the Dietary Guidelines for the Brazilian Population. Thus, the assertiveness of the Guidelines’ multidimensional recommendations and the publication of protocols (31) to support health professionals were emphasized.

Some limitations should be noted. Because only adult users of Primary Health Care services in Goiânia were included in the study, the results cannot be extrapolated to other age groups and regions of the country, given the broad cultural and dietary diversity. The level of physical activity may not accurately reflect its intensity, as it is based on participants’ self-perception. Another limitation was the use of self-reported weight and height measurements for part of the sample, although measured measurements show high agreement with self-reported measurements (32). In this study, the nutritional status variable was categorized, which helped minimize potential discrepancies, since small absolute differences in measurements tend not to alter the classification of individuals into body mass index categories (33). Although analysis stratified by sex was used to reduce bias, the differentiated loss of men in the sample may have compromised the representativeness of the findings. This difference may be related to the lower engagement of men in health studies (34).

The strengths of this study included conducting a pilot study, collecting data in all of Goiânia’s health districts, and using the Food and Nutrition Surveillance System Food Consumption Markers Form, adopted nationwide. The internal structure of this instrument proved stable in terms of evidence of measurement invariance across Brazilian macroregions, life stages, and over time (35). Furthermore, studies evaluating dietary practices (7,16), especially those related to commensality, are scarce in the literature.

The conclusion reached is that adherence to the commensality practices recommended by the Dietary Guidelines, such as eating at the table, in company, and without looking at screens, are associated with healthier food consumption among adult female Primary Health Care service users in Goiânia. These associations were not observed in men. Including these recommendations in health promotion initiatives within Primary Health Care can contribute to improving the quality of the Brazilian population’s food intake.
