# Erratum: "Association between commensality practices and healthy food consumption in Primary Care: a cross-sectional study, Goiânia, 2022-2023”

**DOI:** 10.1590/S2237-96222025v34e20240367e.en

**Published:** 2026-09-14

In the article "Association between commensality practices and healthy food consumption in Primary Care: a cross-sectional study, Goiânia, 2022-2023", doi 10.1590/S2237-96222025v34e20240367.en, published on *Epidemiol Serv Saude*. 2025:34;e20240367, on page 1.

**Original text:**

"Received: 30/8/2024 Approved: 18/6/2024"

**Corrected text:**

"Received: 30/8/2024 Approved: 18/6/2025"

