# Supplementary material for: Association between commensality practices and healthy food consumption in Primary Care: a cross-sectional study, Goiânia, 2022-2023
Source: Epidemiol Serv Saude. 2025 Oct 27;34:e20240367. doi: 10.1590/S2237-96222025v34e20240367.en (PMC12560227; doi:10.1590/S2237-96222025v34e20240367.en)
Supplement: Supplementary Table 1 [file 2237-9622-ress-34-e20240367-supp01-en.pdf]

Supplementary Table 1. Sociodemographic and behavioral characteristics according to the eating habits of adult Primary Health Care service users in Goiânia, Goiás, Brazil, 2022-2023 (n=783)

**Variables**	**Score for recommended commensality practices** (n=783)		**Habit of having screen-free meals** (n=783)		**Habit of having eating food at the table** (n=783)		**Habit of having eating food with company** (n=783)	
	**Median (IQR)**	**p-value**	**Yes n (%)**	**p-value**	**Yes n (%)**	**p-value**	**Yes n (%)**	**p-value**
**Total sample**[f]	2 (1-3)	-	307 (39.2)	-	490 (62.6)	-	605 (77.3)	-
**Age** (years)[a] (n=779)	-	-	41 (30-50)	<0.001[b]	39 (29-48)	0.002[b]	37 (28-46)	0.762[a]
18-35	2 (1-2)	<0.001[b]	106 (34.6)	<0.001[c]	192 (39.3)	0.006[c]	258 (42.6)	0.706[b]
36-59	2 (1-3)		200 (65.4)		297 (60.7)		347 (57.4)	
**Sex** (n=783)								
Female	2 (1-3)	0.694[b]	258 (84.0)	0.159[c]	394 (80.4)	0.262[c]	497 (82.1)	0.472[b]
Male	2 (1-2)		49 (16.0)		96 (19.6)		108 (17.8)	
**Race/skin color** (n=781)								
White	2 (1-3)a	0.003[d]	66 (21.5)	0.370[c]	114 (23.3)	<0.001[c]	130 (21.5)	0.498[b]
Black	2 (1-2)b		42 (13.7)		62 (12.7)		81 (13.4)	
Mixed race	2 (1-3)a		190 (61.9)		302 (61.8)		368 (60.9)	
Asian	1 (1-2)c		9 (2.9)		11 (2.2)		25 (4.1)	
**Schooling level** (n=781)								
Up to complete elementary	2 (1-3)	0.292[d]	112 (36.6)	0.260[c]	158 (32.3)	0.055[c]	210 (34.8)	0.571[b]
Complete high school	2 (1-3)		146 (47.7)		247 (50.5)		306 (50.7)	
Complete higher education	2 (1-3)		48 (15.7)		84 (17.2)		88 (14.6)	
**Monthly Family income** (minimum wages) (n=747)								
<1	2 (1-2)	0.129[d]	18 (6.2)	0.787[c]	32 (6.9)	0.001[c]	43 (7.4)	0.329[b]
1	2 (1-3)		87 (29.9)		125 (26.8)		170 (29.4)	
2-3	2 (1-3)		130 (44.7)		208 (44.6)		270 (46.6)	
4-5	2 (1-3)		33 (11.3)		57 (12.2)		56 (9.7)	

Variables	Score for recommended commensality practices (n=783)		Habit of having screen-free meals (n=783)		Habit of having eating food at the table (n=783)		Habit of having eating food with company (n=783)	
	**Median (IQR)**	**p-value**	**Yes n (%)**	**p-value**	**Yes n (%)**	**p-value**	**Yes n (%)**	**p-value**
>5	2 (1-3)		23 (7.9)		44 (9.4)		40 (6.9)	
**Body Mass Index (kg/m²)**[a]	-	-	26,5 (23.5-30.8)	0.267[b]	26.40 (23.3-30.3)	0.477[b]	26.13 (23.1-30.3)	0.517[b]
**Nutritional Status** (n=761)								
Underweight	2 (1-2)	0.982[c]	7 (2.3)	0.430[c]	18 (3.8)	0.908[c]	23 (3.9)	0.865[d]
Healthy weight	2 (1-3)		113 (37.8)		182 (38.1)		221 (37.6)	
Overweight	2 (1-3)		98 (32.8)		145 (30.3)		186 (31.7)	
Obese	2 (1-3)		81 (27.1)		133 (27.8)		157 (26.7)	
**Tobacco use** (n=747)								
Yes	1 (1-2)	<0.001[b]	24 (8.1)	0.054[c]	33 (7.0)	<0.001[c]	51 (8.8)	0.001[c]
No	2 (1-3)		271 (91.9)		435 (92.9)		525 (91.1)	
**Alcohol use** (n=747)								
Yes	2 (1-2)	0.330[b]	100 (33.8)	0.035[c]	188 (40.2)	0.203[c]	216 (37.5)	0.343[c]
No	2 (1-3)		196 (66.2)		280 (59.8)		360 (62.5)	
**Physical activity level** (n=757)								
Sedentary	2 (1-2)	<0.001[e]	140 (46.8)	0.005[c]	214 (45.2)	0.000[c]	293 (50.2)	0.059[c]
Moderate	2 (1-3)		91 (30.4)		134 (28.4)		148 (25.3)	
Active	2 (1-3)		58 (19.4)		111 (23.5)		129 (22.1)	
Very active	2 (1-2)		10 (3.3)		13 (2.7)		14 (2.4)	

Notes: [a]Values presented as medians (interquartile range). [b] Mann-Whitney U test; [c]Pearson's chi-square test; [d]Fisher's exact test; [e]Kruskal-Wallis test; [f]Some variables had n<783 due to missing data.
