# Supplementary material for: Association between commensality practices and healthy food consumption in Primary Care: a cross-sectional study, Goiânia, 2022-2023
Source: Epidemiol Serv Saude. 2025 Oct 27;34:e20240367. doi: 10.1590/S2237-96222025v34e20240367.en (PMC12560227; doi:10.1590/S2237-96222025v34e20240367.en)
Supplement: Tabela Suplementar 1 [file 2237-9622-ress-34-e20240367-supp01-pt.pdf]

Tabela Suplementar 1. Características sociodemográficas e comportamentais segundo as práticas alimentares de adultos usuários da Atenção Primária à Saúde de Goiânia, Goiás, Brasil, 2022-2023 (n=783)

**Variáveis**	**Escore de práticas de comensalidade recomendadas** (n=783)		**Hábito de realizar as refeições sem o uso das telas** (n=783)		**Hábito de consumir alimentos à mesa** (n=783)		**Hábito de consumir alimentos com companhia** (n=783)	
	**Mediana (IIQ)**	**p-valor**	**Sim n (%)**	**p-valor**	**Sim n (%)**	**p-valor**	**Sim n (%)**	**p-valor**
**Amostra total**[f]	2 (1-3)	-	307 (39,2)	-	490 (62,6)	-	605 (77,3)	-
**Idade** (anos)[a] (n=779)	-	-	41 (30-50)	<0,001[b]	39 (29-48)	0,002[b]	37 (28-46)	0,762[a]
18-35	2 (1-2)	<0,001[b]	106 (34,6)	<0,001[c]	192 (39,3)	0,006[c]	258 (42,6)	0,706[b]
36-59	2 (1-3)		200 (65,4)		297 (60,7)		347 (57,4)	
**Sexo** (n=783)								
Feminino	2 (1-3)	0,694[b]	258 (84,0)	0,159[c]	394 (80,4)	0,262[c]	497 (82,1)	0,472[b]
Masculino	2 (1-2)		49 (16,0)		96 (19,6)		108 (17,8)	
**Raça/cor da pele** (n=781)								
Branca	2 (1-3)a	0,003[d]	66 (21,5)	0,370[c]	114 (23,3)	<0,001[c]	130 (21,5)	0,498[b]
Preta	2 (1-2)b		42 (13,7)		62 (12,7)		81 (13,4)	
Parda	2 (1-3)a		190 (61,9)		302 (61,8)		368 (60,9)	
Amarela	1 (1-2)c		9 (2,9)		11 (2,2)		25 (4,1)	
**Nível de escolaridade** (n=781)								
Até fundamental completo	2 (1-3)	0,292[d]	112 (36,6)	0,260[c]	158 (32,3)	0,055[c]	210 (34,8)	0,571[b]
Médio completo	2 (1-3)		146 (47,7)		247 (50,5)		306 (50,7)	
Superior completo	2 (1-3)		48 (15,7)		84 (17,2)		88 (14,6)	
**Renda mensal familiar** (salários-mínimos) (n=747)								
<1	2 (1-2)	0,129[d]	18 (6,2)	0,787[c]	32 (6,9)	0,001[c]	43 (7,4)	0,329[b]
1	2 (1-3)		87 (29,9)		125 (26,8)		170 (29,4)	
2-3	2 (1-3)		130 (44,7)		208 (44,6)		270 (46,6)	

Variáveis	Escore de práticas de comensalidade recomendadas (n=783)		Hábito de realizar as refeições sem o uso das telas (n=783)		Hábito de consumir alimentos à mesa (n=783)		Hábito de consumir alimentos com companhia (n=783)	
	**Mediana (IIQ)**	**p-valor**	**Sim n (%)**	**p-valor**	**Sim n (%)**	**p-valor**	**Sim n (%)**	**p-valor**
4-5	2 (1-3)		33 (11,3)		57 (12,2)		56 (9,7)	
>5	2 (1-3)		23 (7,9)		44 (9,4)		40 (6,9)	
**Índice de massa corporal (kg/m²)[a]**	-	-	26,5 (23,5-30,8)	0,267[b]	26,40 (23,3-30,3)	0,477[b]	26,13 (23,1-30,3)	0,517[b]
**Estado nutricional** (n=761)								
Baixo peso	2 (1-2)	0,982[e]	7 (2,3)	0,430[c]	18 (3,8)	0,908[c]	23 (3,9)	0,865[d]
Eutrofia	2 (1-3)		113 (37,8)		182 (38,1)		221 (37,6)	
Sobrepeso	2 (1-3)		98 (32,8)		145 (30,3)		186 (31,7)	
Obesidade	2 (1-3)		81 (27,1)		133 (27,8)		157 (26,7)	
**Uso de tabaco** (n=747)								
Sim	1 (1-2)	<0,001[b]	24 (8,1)	0,054[c]	33 (7,0)	<0,001[c]	51 (8,8)	0,001[c]
Não	2 (1-3)		271 (91,9)		435 (92,9)		525 (91,1)	
**Uso de álcool** (n=747)								
Sim	2 (1-2)	0,330[b]	100 (33,8)	0,035[c]	188 (40,2)	0,203[c]	216 (37,5)	0,343[c]
Não	2 (1-3)		196 (66,2)		280 (59,8)		360 (62,5)	
**Nível de atividade física** (n=757)								
Sedentário	2 (1-2)	<0,001[e]	140 (46,8)	0,005[c]	214 (45,2)	0,000[c]	293 (50,2)	0,059[c]
Moderado	2 (1-3)		91 (30,4)		134 (28,4)		148 (25,3)	
Ativo	2 (1-3)		58 (19,4)		111 (23,5)		129 (22,1)	
Muito ativo	2 (1-2)		10 (3,3)		13 (2,7)		14 (2,4)	

Notas: [a]Valores apresentados em mediana (variação interquartil). [b]Teste de U de Mann-Whitney; [c]Teste qui-quadrado de Pearson; [d]Teste exato de Fisher; [e]Teste Kruskal-Wallis; [f]Algumas variáveis apresentam n<783 devido aos dados faltantes.
