# Supplementary material for: Association between commensality practices and healthy food consumption in Primary Care: a cross-sectional study, Goiânia, 2022-2023
Source: Epidemiol Serv Saude. 2025 Oct 27;34:e20240367. doi: 10.1590/S2237-96222025v34e20240367.en (PMC12560227; doi:10.1590/S2237-96222025v34e20240367.en)
Supplement: Translation

# Translation: Associação entre práticas de comensalidade e consumo alimentar saudável na Atenção Primária: estudo transversal, Goiânia, 2022-2023

**DOI:** 10.1590/S2237-96222025v34e20240367.pt

**Authors:** Roberta Sena Reis, Raquel Machado Schincaglia, Mariana Martins Moreira, Jordanna de Souza Ferreira, Priscila Farage de Gouveia, Andréa Sugai Mortoza, Marilia Mendonça Guimarães, Maria do Rosário Gondim Peixoto, Luciana Bronzi de Souza

**Affiliations:** 1 Universidade Federal de Goiás, Faculdade de Nutrição, Goiânia, GO, Brasil

**Keywords:** Ingestão de Alimentos, Guias Alimentares, Atenção Primária à Saúde, Adulto, Estudos Transversais

## Abstract

**Objetivo:**

Verificar a associação entre a adesão às práticas de comensalidade recomendadas pelo *Guia Alimentar para População Brasileira* e o consumo alimentar saudável.

**Métodos:**

Trata-se de estudo transversal, com adultos da Atenção Primária à Saúde de Goiânia. O escore de consumo alimentar saudável (0-7 pontos) foi construído atribuindo 1 ponto para cada “sim” reportado ao consumo de três marcadores de alimentos saudáveis e para cada “não” reportado ao consumo de quatro marcadores de alimentos não saudáveis. O escore de práticas de comensalidade recomendadas (0-3 pontos) foi avaliado pela soma de hábitos de realizar refeições sem uso das telas, consumir alimentos à mesa e comer em companhia. Foram avaliadas variáveis sociodemográficas e comportamentais. Realizou-se regressão quantílica, com significância de 5%.

**Resultados:**

Avaliaram-se 783 adultos, maioria pardos, mulheres, com excesso de peso. O feijão foi o marcador saudável (74,8%), e as bebidas adoçadas, o marcador não saudável (52,1%) mais consumidos no dia anterior. Comer em companhia (77,3%) foi a prática de comensalidade mais prevalente. A mediana de escore de consumo alimentar saudável foi 5 (4-6), e a de práticas de comensalidade recomendadas foi 2 (1-3) pontos. Entre as mulheres, para cada aumento de uma prática de comensalidade recomendada realizada, houve aumento do escore de consumo alimentar saudável em 0,42 (intervalo de confiança de 95% [IC95%] 0,18; 0,66) no percentil 10; 0,31 (IC95% 0,13; 0,49) no percentil 25; 0,17 (IC95% 0,04; 0,29) no percentil 50; e 0,16 (IC95% 0,02-0,31) no percentil 75, mas não se associou ao percentil 90. Não foram encontradas associações entre homens.

**Conclusão:**

Práticas de comensalidade recomendadas pelo Guia associaram-se ao consumo alimentar mais saudável na amostra de mulheres adultas da Atenção Primária.

**Aspectos éticos**

Esta pesquisa respeitou os princípios éticos, obtendo os seguintes dados de aprovação:

Comitê de ética em pesquisa: Universidade Federal de Goiás

Número do parecer: 5.213.102

Data de aprovação: 26/1/2022

Certificado de apresentação de apreciação ética: 26118319.5.0000.5083

Registro do consentimento livre e esclarecido: Obtido de todos os participantes antes da coleta.

## Introdução 

As práticas alimentares são compreendidas como o conjunto de atividades alimentares, que incluem o planejamento das refeições, o modo de alimentar-se e o consumo alimentar em si (1). Essas práticas estão relacionadas a aspectos individuais, como os significados, os valores e as representações simbólicas atribuídos à comida, assim como ao ambiente alimentar (1,2).

As práticas alimentares recomendadas pelo *Guia Alimentar para População Brasileira* (2014) abrangem a comensalidade (3) – com origem no latim “mensa”, que significa “compartilhar a mesa” – e o foco no modo em que se come, não apenas no que se come (4). Três práticas de comensalidade são apresentadas: comer com regularidade e atenção, comer em ambientes apropriados e comer em companhia. A adoção dessas práticas torna o momento da refeição prazeroso e coletivo. 

O Guia aborda o contexto em que a alimentação é realizada e apresenta recomendações de consumo alimentar baseadas na classificação Nova (não é um acrônimo). Esta categoriza os alimentos conforme a extensão e a finalidade do processamento. As orientações estão direcionadas aos aspectos não quantitativos da alimentação, com foco nos alimentos e grupos de alimentos e em como esses produzem ou não saúde (3). 

Apesar do avanço dessas recomendações alimentares, verificou-se, no Brasil, aumento da participação de ultraprocessados nas calorias totais disponíveis nos domicílios (5). Esse cenário destaca a necessidade de monitorar a relação entre práticas alimentares e a qualidade da dieta, já que essa baixa qualidade pode aumentar o risco para doenças crônicas não transmissíveis (6). As práticas de comensalidade se associam diretamente ao consumo de alimentos in natura ou minimamente processados e inversamente ao consumo de processados e ultraprocessados (7). No contexto da Atenção Primária à Saúde, estudos que avaliem as práticas de comensalidade são necessários para identificar potencialidades e dificuldades no território para a promoção da alimentação adequada e saudável. É nesse lócus onde os maiores esforços de promoção e proteção às práticas saudáveis, entre elas a alimentação, devem ser concentrados. 

Este estudo teve como objetivo verificar a associação entre a adesão às práticas de comensalidade recomendadas pelo Guia e o consumo alimentar saudável entre adultos usuários da Atenção Primária à Saúde.

## Métodos 

### Desenho do estudo 

Trata-se de estudo transversal relacionado à pesquisa matriz “Ambiente alimentar, da atividade física e fatores associados em indivíduos adultos residentes em município de grande porte”. Este estudo objetivou investigar a existência de associação entre o ambiente alimentar e da prática de atividade física percebidos, as práticas alimentares, o estado nutricional e o estado de segurança alimentar em adultos de Goiânia. 

### Contexto do estudo

Goiânia é a capital de Goiás. Conforme o Censo Demográfico de 2022, a cidade possui área geográfica de 729,296 km^2^ e 1.437.366 habitantes. Em 2010, esse município apresentou índice de desenvolvimento humano municipal alto (0,799) (8). Segundo a página e-Gestor, a cobertura média de Atenção Primária de Goiânia, em 2022, foi 51,0% e sua organização ocorreu em sete distritos sanitários (Norte, Sul, Oeste, Leste, Sudoeste, Campinas-Centro e Noroeste). Em 2021, Goiânia tinha 22 centros de saúde e 61 centros de saúde da família. 

### Participantes 

No estudo matriz, foram incluídos usuários das unidades da Atenção Primária à Saúde selecionadas, de 18-59 anos de idade, de ambos os sexos, que residiam na área de abrangência do distrito. Não se consideraram gestantes nem adultos com amputação/problemas ortopédicos que impediam a antropometria; com problemas psiquiátricos/neurológicos que inviabilizassem a entrevista; e que estavam fazendo tratamento oncológico.

### Tamanho da amostra

O cálculo amostral foi realizado na plataforma OpenEpi, considerando prevalência de 50,0% para os desfechos do estudo matriz, erro absoluto de 5,0%, nível de confiança de 95,0%, efeito de delineamento de 1,5 e população estimada de 876.794 adultos (com base no Censo Demográfico 2022 e na projeção de 61,0% de adultos em Goiânia) (9). Obteve-se amostra mínima de 576 indivíduos, a qual foi acrescida 20,0%, com amostra final necessária de 691 indivíduos. 

A coleta de dados da pesquisa matriz finalizou em um ano, com 968 indivíduos entrevistados, distribuídos de forma proporcional ao número de habitantes dos distritos sanitários. Foram excluídos dois indivíduos por questionários em branco e 183 que não responderam às questões relacionadas às práticas de comensalidade e/ou aos marcadores de consumo alimentar, resultando em 783 indivíduos. Foram comparados os 183 indivíduos excluídos com os 783 incluídos, e evidenciou-se diferença significativa na proporção de indivíduos do sexo masculino, sendo 25,68% entre os excluídos e 18,39% entre os incluídos (p-valor 0,030). Não foram identificadas diferenças para idade, raça/cor da pele, escolaridade, renda mensal familiar, estado nutricional, uso de tabaco e álcool e atividade física. 

### Coleta de dados

Os participantes foram aleatoriamente selecionados na sala de espera enquanto aguardavam atendimento na unidade da Atenção Primária à Saúde. 

A coleta de dados foi realizada entre maio de 2022 e maio de 2023, presencialmente, em 13 unidades (1 centro de saúde e 12 centros de saúde da família), distribuídas nos sete distritos sanitários. Em cada distrito, selecionaram-se duas unidades com maior número de atendimentos. No distrito Sul, existiam duas unidades possíveis de seleção e uma delas recusou participação. 

O cronograma de coleta de dados foi definido com base nos sete distritos sanitários, de forma a abranger um distrito por semana. Um sorteio era realizado a cada sete semanas para definir a ordem em que esses distritos seriam visitados. Após a finalização de um ciclo de sete semanas, um novo sorteio era realizado para garantir aleatoriedade e sazonalidade da coleta de dados. Os ciclos foram repetidos até a obtenção da amostra prevista para cada distrito. 

A coleta de dados foi realizada por meio da aplicação de questionário estruturado e padronizado. Foi realizado estudo-piloto para testar métodos e instrumentos, e os respondentes foram incluídos na amostra do estudo. 

### Variáveis 

O desfecho do estudo foi o escore de marcadores de consumo alimentar, construído com base no instrumento do Sistema de Vigilância Alimentar e Nutricional (10). Esse instrumento considerou o consumo no dia anterior de três grupos de alimentos saudáveis – feijão; frutas frescas (exceto suco de frutas); e hortaliças (exceto batata, mandioca, aipim, macaxeira, cará e inhame) – e de quatro grupos de alimentos não saudáveis – hambúrguer e/ou embutidos (presunto, mortadela, salame, linguiça, salsicha); bebidas adoçadas (refrigerante, suco de caixinha, suco em pó, água de coco em caixinha, xaropes de guaraná/groselha, suco de fruta com adição de açúcar); macarrão instantâneo, salgadinhos de pacote ou bolacha salgada (bolacha água e sal); e bolacha recheada, doces/guloseimas (balas, pirulitos, chiclete, caramelo, gelatina). Os entrevistados poderiam responder sim ou não para cada grupo. 

Na avaliação do escore, foi atribuído 1 ponto para cada “sim” reportado ao consumo de cada um dos três grupos de alimentos saudáveis e para cada “não” relatado para o consumo de cada um dos quatros grupos de alimentos não saudáveis. O escore de cada participante variou de 0-7 pontos, de forma que, quanto maior a pontuação, mais saudável o consumo alimentar. O escore de marcadores de consumo alimentar utilizado baseou-se em Louzada et al. (11), que avaliaram os marcadores de consumo saudável (0 a 3) e não saudável (0 a 4) na perspectiva de escore, e esse apresentou bom potencial para refletir a qualidade geral da dieta. Neste estudo, trabalhou-se na mesma perspectiva, embora com categoria combinada de respostas para os marcadores de consumo alimentar (variando de 0 a 7).

As variáveis de exposição foram as práticas de comensalidade recomendadas pelo Guia: ter o hábito de realizar as refeições sem o uso das telas, incluindo TV, computador e/ou celular (sim, não); ter o hábito de se alimentar/comer à mesa (sim, não) e ter o hábito de se alimentar junto à família ou às pessoas que residem com você (sim, não). Para cada “sim” reportado, foi atribuído 1 ponto. Os pontos foram somados, e o escore de cada participante variou de 0 a 3. Quanto maior a pontuação, mais práticas de comensalidade recomendadas foram realizadas.

As variáveis independentes foram: idade (anos completos); sexo (masculino, feminino); raça/cor da pele (branca, preta, parda, amarela); nível de escolaridade (até ensino fundamental completo, ensino médio completo e ensino superior completo); renda mensal familiar, em salário-mínimo mínimo à época (<1, 1, 2-3, 4-5, >5); índice de massa corporal (baixo peso <18,5; eutrofia 18,5-24,9; sobrepeso 25,0-29,9; obesidade ≥30,0) (12); uso de tabaco (sim, não); uso de álcool (sim, não); e nível de atividade física percebido (sedentário, moderado, ativo, muito ativo).

O índice de massa corporal foi calculado considerando peso (quilogramas) dividido pela estatura (metro) ao quadrado (12). O peso e a estatura foram aferidos ou referidos (considerando seu último peso), conforme disponibilidade de equipamento no dia da coleta de dados. 

### Análise estatística

O banco de dados foi construído no programa Epi Info, versão 7.0 com dupla entrada. A análise dos dados foi realizada pelo Stata, versão 12.0, e estes foram apresentados em frequências absolutas (n) e relativas (%) ou mediana e variação interquartil (p25-p75). Dadas as características discretas ou não normais das variáveis numéricas, realizou-se teste de U de Mann-Whitney ou Kruskal-Wallis para comparação entre grupos. Testes do qui-quadrado de Pearson ou exato de Fisher foram usados para comparar proporções entre grupos. Foi calculada a correlação de Spearman (ρ). 

Aplicaram-se regressões quantílicas bruta e ajustada para a amostra total e estratificada por sexo, usando como desfecho o escore de consumo alimentar, como exposição às práticas de comensalidade recomendadas, e como variáveis de ajuste as variáveis idade, sexo (apenas para amostra total), escolaridade, índice de massa corporal, nível de atividade física percebido, uso de álcool e uso de tabaco. A regressão foi realizada utilizando os percentis 10, 25, 50, 75 e 90 do escore de consumo alimentar saudável. Essa análise foi escolhida, pois permitiu avaliar mudanças na distribuição em diferentes percentis, quando não houve normalidade no desfecho. O tamanho do efeito não padronizado (t) foi calculado pela razão entre o coeficiente (𝛽) e o erro-padrão (SE) da regressão quantílica ajustada. Adotou-se o nível de significância de 5%.

## Resultados 

Dos 783 participantes, a maioria tinha idade entre 36 e 59 anos (n=444; 57,0%), era do sexo feminino (n=639; 81,6%) e autodeclarada parda (n=468; 59,9%), tinha ensino médio completo (n=401; 51,3%), renda mensal familiar de até 3 salários mínimos (n=616; 82,5%), excesso de peso (n=446; 58,6%), não usava tabaco (n=666; 89,2%), não consumia álcool (n=460; 61,6%) e tinha autopercepção de nível de atividade física classificado como sedentário (n=398; 52,6%) (Tabela 1).

**Tabela 1:** Características sociodemográficas e comportamentais segundo marcadores de consumo alimentar saudável de adultos usuários da Atenção Primária à Saúde. Goiânia, 2022-2023 (n=783)

Variáveis	Amostra total (n=783)	Marcadores saudáveis								
		Feijão (n=783)			Frutas frescas (n=783)			Hortaliças (n=783)		
	n (%)	Sim n (%)	Não n (%)	p-valor	Sim n (%)	Não n (%)	p-valor	Sim n (%)	Não n (%)	p-valor
**Amostra total^f^**	-	586 (74,8)	197 (25,2)	-	468 (59,8)	315 (40,2)	-	545 (69,6)	238 (30,4)	-
**Idade** (**anos)a** (n=779)				0,146^c^			0,001^c^			0,002^c^
18-35	335 (43,0)	242 (41,5)	93 (47,5)		177 (38,1)	158 (50,3)		213 (39,3)	122 (51,5)	
36-59	444 (57,0)	341 (58,5)	103 (52,5)		288 (61,9)	156 (49,7)		329 (60,7)	115 (48,5)	
**Sexo** (n=783)				0,961^c^			0,354^c^			0,450^c^
Feminino	639 (81,6)	478 (81,6)	161 (81,7)		377 (80,6)	262 (83,2)		441 (80,9)	198 (83,2)	
Masculino	144 (18,4)	108 (18,4)	36 (18,3)		91 (19,4)	53 (16,8)		104 (19,1)	40 (16,8)	
**Raça/cor da pele** (n=781)				0,033^d^			0,002^c^			0,514^c^
Branca	167 (21,4)	114 (19,5)	53 (26,9)		101 (21,6)	66 (21,0)		118 (21,7)	49 (20,6)	
Preta	111 (14,2)	80 (13,7)	31 (15,8)		66 (14,1)	45 (14,3)		72 (13,3)	39 (16,4)	
Parda	468 (59,9)	359 (61,5)	109 (55,3)		290 (62,1)	178 (56,7)		331 (61,0)	137 (57,6)	
Amarela	35 (4,5)	31 (5,3)	4 (2,0)		10 (2,2)	25 (8,0)		22 (4,0)	13 (5,4)	
**Escolaridade** (n=781)				0,076^c^			0,017^c^			0,062^c^
Até fundamental completo	264 (33,8)	200 (34,2)	64 (32,5)		149 (31,9)	115 (36,6)		173(31,8)	91 (38,2)	
Médio completo	401 (51,3)	307 (52,6)	94 (47,7)		235 (50,3)	166 (52,9)		280(51,6)	121 (50,9)	
Superior completo	116 (14,9)	77 (13,2)	39 (19,8)		83 (17,8)	33 (10,5)		90(16,6)	26 (10,9)	
**Renda familiar mensal** (salários-mínimos) (n=747)				0,610^c^			0,021^c^			0,007^c^
<1	55 (7,4)	42 (7,5)	13 (6,9)		31 (6,9)	24 (8,0)		32 (6,1)	23 (10,1)	
1	227 (30,4)	175 (31,4)	52 (27,4)		120 (26,8)	107 (35,7)		146 (28,1)	81 (35,7)	
2-3	334 (44,7)	249 (44,7)	85 (44,7)		207 (46,3)	127 (42,3)		237 (45,6)	97 (42,7)	
4-5	77 (10,3)	53 (9,5)	24 (12,6)		48 (10,7)	29 (9,7)		60 (11,5)	17 (7,5)	
>5	54 (7,2)	38 (6,8)	16 (8,4)		41 (9,2)	13 (4,3)		45 (8,6)	9 (4,0)	
**Índice de massa corporal** (kg/m^2^)	26,2 (23,1-30,4)^a^	27,0 (5,8)	27,8 (6,0)	0,094^b^	27,2 (5,8)	27,2 (6,0)	0,956^b^	27,4 (6,0)	26,6 (5,7)	0,093^b^
**Estado nutricional** (n=761)				0,214^d^			0,848^c^			0,839^c^
Baixo peso	28 (3,7)	25 (4,4)	3 (1,6)		16 (3,5)	12 (4,0)		20 (3,8)	8 (3,5)	
Eutrofia	287 (37,7)	221 (38,6)	66 (35,1)		176 (38,5)	111 (36,5)		194 (36,7)	93 (40,1)	
Sobrepeso	238 (31,3)	176 (30,7)	62 (33,0)		138 (30,2)	100 (32,9)		169 (31,9)	69 (29,7)	
Obesidade	208 (27,3)	151 (26,3)	57 (30,3)		127 (27,8)	81 (26,6)		146 (27,6)	62 (26,7)	
**Uso de tabaco** (n=747)				0,868^c^			<0,001^c^			0,032^c^
Sim	81 (10,8)	60 (10,7)	21 (11,2)		31 (6,9)	50 (16,7)		48 (9,2)	33 (14,5)	
Não	666 (89,2)	499 (89,3)	167 (88,8)		417 (93,1)	249 (83,3)		472 (90,8)	194 (85,5)	
**Uso de álcool** (n=747)				0,078^c^			<0,001^c^			0,770^c^
Sim	287 (38,4)	205 (36,6)	82 (43,9)		145 (32,4)	142 (47,5)		198 (38,1)	89 (39,2)	
Não	460 (61,6)	355 (63,4)	105 (56,1)		303 (67,6)	157 (52,5)		322 (61,9)	138 (60,8)	
**Atividade física** (n=757)				0,637^d^			<0,001^d^			0,004^d^
Sedentário	398 (52,6)	299 (52,4)	99 (52,9)		202 (44,8)	196 (64,1)		256 (48,6)	142 (61,7)	
Moderado	182 (24,0)	140 (24,6)	42 (22,5)		122 (27,0)	60 (19,6)		131 (24,9)	51 (22,2)	
Ativo	157 (20,7)	114 (20,0)	43 (23,0)		111 (24,6)	46 (15,0)		124 (23,5)	33 (14,4)	
Muito ativo	20 (2,6)	17 (3,0)	3 (1,6)		16 (3,6)	4 (1,3)		16 (3,0)	4 (1,7)	

^a^Valores apresentados em mediana (variação interquartil); ^b^Teste de U de Mann-Whitney; ^c^Teste qui-quadrado de Pearson; ^d^Teste exato de Fisher; ^e^Teste Kruskal-Wallis; ^f^Algumas variáveis apresentam n<783 devido aos dados faltantes.

Quanto aos marcadores de consumo alimentar saudável, 74,8% (n=586) dos participantes reportaram ter consumido feijão no dia anterior; 59,8% (n=468), frutas frescas; e 69,6% (n=545), hortaliças (Tabela 1). Em relação aos marcadores de consumo não saudável, bebidas adoçadas foi o marcador mais frequentemente consumido no dia anterior (n=408; 52,1%). Houve menor frequência de consumo de bolacha recheada, doces ou guloseimas (n=224; 28,6%), hambúrguer e/ou embutidos (n=152; 19,4%) e macarrão instantâneo, salgadinhos de pacote ou bolacha salgada (n=143; 18,3%) (Tabela 2). As diferenças nos marcadores de consumo alimentar, segundo variáveis sociodemográficas e comportamentais, encontram-se nas Tabelas 1 e 2. O escore de consumo alimentar saudável apresentou mediana de 5 (4-6) pontos na amostra total e diferiu segundo idade (p-valor<0,001), sexo (p-valor 0,032), uso de tabaco (p-valor 0,005), uso de álcool (p-valor<0,001) e nível de atividade física (p-valor=0,002) (Tabela 2).

**Tabela 2:** Características sociodemográficas e comportamentais segundo marcadores de consumo alimentar não saudável de adultos usuários da Atenção Primária à Saúde. Goiânia, 2022-2023 (n=783)

Variáveis	Marcadores não saudáveis												Escore de consumo alimentar saudável (n=783)	
	Hambúrguer e/ou embutidos (n=783)			Bebidas adoçadas (n=783)			Macarrão instantâneo, salgadinhos de pacote ou bolacha salgada (n=783)			Bolacha recheada, doces ou guloseimas (n=783)				
	Sim n (%)	Não n (%)	p-valor	Sim n (%)	Não n (%)	p-valor	Sim n (%)	Não n (%)	p-valor	Sim n (%)	Não n (%)	p-valor	Mediana (IIQ)	p-valor
**Amostra total^f^**	152 (19,4)	631 (80,6)	-	408 (52,1)	375 (47,9)	-	143 (18,3)	640 (81,7)	-	224 (28,6)	559 (71,4)	-	5 (4-6)	-
**Idade (anos)^a^** (n=779)			0,013^c^			<0,001^c^			<0,001^c^			<0,001^c^		<0,001^b^
18-35	79 (52,0)	256 (40,8)		210 (51,7)	125 (33,5)		82 (57,3)	253 (39,8)		128 (57,7)	207 (37,2)		4 (3-6)	
36-59	73 (48,0)	371 (59,2)		196 (48,3)	248 (66,5)		61 (42,7)	383 (60,2)		94 (42,3)	350 (62,8)		5 (4-6)	
**Sexo** (n=783)			0,005^c^			0,002^c^			0,173^c^			0,016^c^		0,032^b^
Feminino	112 (73,7)	527 (83,5)		316 (77,4)	323 (86,1)		111 (77,6)	528 (82,5)		171 (76,3)	468 (83,7)		5 (4-6)	
Masculino	40 (26,3)	104 (16,5)		92 (22,6)	52 (13,9)		32 (22,4)	112 (17,5)		53 (23,7)	91 (16,3)		5 (3,5-6)	
**Raça/cor da pele** (n=781)			0,188^d^			0,276^c^			0,802^d^			0,978^c^		0,631^e^
Branca	25 (16,4)	142 (22,6)		95 (23,3)	72 (19,3)		31 (21,7)	136 (21,3)		47 (21,1)	120 (21,5)		5 (4-6)	
Preta	25 (16,4)	86 (13,7)		63 (15,5)	48 (12,8)		21 (14,7)	90 (14,1)		30 (13,4)	81 (14,5)		5 (4-6)	
Parda	98 (64,5)	370 (58,8)		232 (57)	236 (63,1)		87 (60,8)	381 (59,7)		136 (61,0)	332 (59,5)		5 (4-6)	
Amarela	4 (2,6)	31 (4,9)		17 (4,2)	18 (4,8)		4 (2,8)	31 (4,9)		10 (4,5)	25 (4,5)		5 (4-6)	
**Escolaridade** (n=781)			0,495^c^			0,782^c^			0,393^c^			0,087^c^		0,247^e^
Até fundamental completo	46 (30,3)	218 (34,7)		133 (32,7)	131 (35,0)		50 (35,0)	214 (33,5)		70 (31,2)	194 (34,8)		5 (4-6)	
Médio completo	80 (52,6)	321 (51,0)		213 (52,3)	188 (50,3)		77 (53,8)	324 (50,8)		128 (57,1)	273 (49,0)		5 (4-6)	
Superior completo	26 (17,1)	90 (14,3)		61 (15,0)	55 (14,7)		16 (11,2)	100 (15,7)		26 (11,6)	90 (16,2)		5 (4-6)	
**Renda familiar mensal** (salários-mínimos) (n=747)			0,076^c^			0,212^c^			0,257^d^			0,787c		0,142^e^
<1	9 (6,2)	46 (7,7)		25 (6,5)	30 (8,3)		9 (6,9)	46 (7,5)		13 (6,1)	42 (7,9)		5 (4-6)	
1	38 (26,0)	189 (31,4)		105 (27,2)	122 (33,8)		44 (33,6)	183 (29,7)		64 (29,9)	163 (30,6)		5 (4-6)	
2-3	69 (47,3)	265 (44,1)		183 (47,4)	151 (41,8)		58 (44,3)	276 (44,8)		95 (44,4)	239 (44,8)		5 (4-6)	
4-5	23 (15,7)	54 (9,0)		44 (11,4)	33 (9,2)		16 (12,2)	61 (9,9)		26 (12,1)	51 (9,6)		5 (4-6)	
>5	7 (4,8)	47 (7,8)		29 (7,5)	25 (6,9)		4 (3,0)	50 (8,1)		16 (7,5)	38 (7,1)		5,5 (4-6)	
**Índice de massa corporal** (kg/m^2^)	27,6 (6,0)	27,1 (5,9)	0,404^c^	27,0 (5,9)	27,3 (5,8)	0,465^b^	26,2 (6,2)	27,4 (5,8)	0,007^b^	26,9 (6,5)	27,3 (5,6)	0,126^b^		-
**Estado nutricional** (n=761)			0,359^c^			0,812^c^			0,120^c^			0,184^c^		0,709^e^
Baixo peso	6 (4,0)	22(3,6)		17 (4,2)	11 (3,0)		9 (6,5)	19 (3,1)		11 (5,0)	17 (3,1)		4 (4-6)	
Eutrofia	56 (37,6)	231 (37,8)		148 (37,0)	139 (38,5)		58 (41,7)	229 (36,8)		92 (42,0)	195 (36,0)		5 (4-6)	
Sobrepeso	39 (26,2)	199 (32,5)		127 (31,8)	111 (30,8)		40 (28,8)	198 (31,8)		60 (27,4)	178 (32,9)		5 (4-6)	
Obesidade	48 (32,2)	160 (26,1)		108 (27,0)	100 (27,7)		32 (23,0)	176 (28,3)		56 (25,6)	152 (28,0)		5 (4-6)	
**Uso de tabaco** (n=747)			0,144^c^			0,952^c^			0,702^c^			0,599^c^		0,005^b^
Sim	21 (14,2)	60 (10,0)		42 (10,9)	39 (10,8)		16 (11,8)	65 (10,6)		25 (11,8)	56 (10,5)		4 (4-6)	
Não	127 (85,8)	539 (90,0)		343 (89,1)	323 (89,2)		120 (88,2)	546 (89,4)		187 (88,2)	479 (89,5)		5 (4-6)	
**Uso de álcool** (n=747)			0,002^c^			0,013^c^			0,884^c^			0,097^c^		<0,001^b^
Sim	73 (49,7)	214 (35,7)		164 (42,7)	123 (33,9)		53 (39,0)	234 (38,3)		91 (43,1)	196 (36,6)		5 (4-6)	
Não	74 (50,3)	386 (64,3)		220 (57,3)	240 (66,1)		83 (61,0)	377 (61,7)		120 (56,9)	340 (63,4)		5 (4-6)	
**Atividade física** (n=757)			0,979^d^			0,175^c^			0,052^d^			0,235^c^		0,002^e^
Sedentário	76 (51,4)	322 (52,9)		220 (56,0)	178 (48,9)		76 (54,3)	322 (52,2)		111 (51,9)	287 (52,9)		5 (4-6)	
Moderado	36 (24,3)	146 (24,0)		91 (23,1)	91 (25,0)		24 (17,1)	158 (25,6)		58 (27,1)	124 (22,8)		5 (4-6)	
Ativo	32 (21,6)	125 (20,5)		71 (18,1)	86 (23,6)		38 (27,1)	119 (19,3)		37 (17,3)	120 (22,1)		5 (4-6)	
Muito ativo	4 (2,7)	16 (2,6)		11 (2,8)	9 (2,5)		2 (1,4)	18 (2,9)		8 (3,7)	12 (2,2)		5 (4-6,5)	

^a^Valores apresentados em mediana (variação interquartil); ^b^Teste de U de Mann-Whitney; ^c^Teste qui-quadrado de Pearson; ^d^Teste exato de Fisher; ^e^Teste Kruskal-Wallis; ^f^Algumas variáveis apresentam n<783 devido aos dados faltantes.

**Tabela 3:** Práticas de comensalidade segundo marcadores de consumo alimentar saudável de adultos usuários da Atenção Primária à Saúde. Goiânia, 2022-2023 (n=783)

Variáveis	Amostra total (n=783)	Marcadores saudáveis								
		Feijão (n=783)			Frutas frescas (n=783)			Hortaliças (n=783)		
	n (%)	Sim n (%)	Não n (%)	p-valor	Sim n (%)	Não n (%)	p-valor	Sim n (%)	Não n (%)	p-valor
**Amostra total^f^**	-	586 (74,8)	197 (25,2)	-	468 (59,8)	315 (40,2)	-	545 (69,6)	238 (30,4)	-
**Hábito de realizar as refeições sem o uso das telas**				0,016^c^			<0,001^c^			0,001^c^
Sim	476 (60,8)	342 (58,4)	134 (68,0)		261 (55,8)	215 (68,2)		311 (57,1)	165 (69,3)	
Não	307 (39,2)	244 (41,6)	63 (32,0)		207 (44,2)	100 (31,8)		234 (42,9)	73 (30,7)	
**Hábito de consumir alimentos à mesa**				0,527^c^			<0,001^c^			0,001^c^
Sim	490 (62,6)	363 (61,9)	127 (64,5)		327 (69,9)	163 (51,8)		361 (66,2)	129 (54,2)	
Não	293 (37,4)	223 (38,1)	70 (35,5)		141 (30,1)	152 (48,2)		184 (33,8)	109 (45,8)	
**Hábito de consumir alimentos com companhia**				0,222^c^			0,145^c^			0,003^c^
Sim	605 (77,3)	459 (78,3)	146 (74,1)		370 (79,1)	235 (74,6)		437 (80,2)	168 (70,6)	
Não	178 (22,7)	127 (21,7)	51 (25,9)		98 (20,9)	80 (25,4)		108 (19,8)	70 (29,4)	
**Escore de práticas de comensalidade recomendadas** (n=783) Mediana (IIQ)	2 (1-3)	2 (1-3)	2 (1-2)	0,156^b^	2 (1-3)	2 (1-2)	<0,001^b^	2 (1-3)	2 (1-2)	<0,001^b^

^a^Valores apresentados em mediana (variação interquartil); ^b^Teste de U de Mann-Whitney; ^c^Teste qui-quadrado de Pearson; ^d^Teste exato de Fisher; ^e^Teste Kruskal-Wallis; ^f^Algumas variáveis apresentam n<783 devido aos dados faltantes.

Observaram-se diferenças da prevalência dos marcadores de consumo alimentar, segundo as práticas de comensalidade (Tabelas 3 e 4). A mediana do escore de práticas de comensalidade recomendadas na amostra total foi 2 (1-3) pontos; 39,2% (n=307) dos adultos relataram o hábito de realizar as refeições sem uso de telas; 62,6% (n=490), o hábito de comer à mesa; e 77,3% (n=605), o comer em companhia. O escore de consumo alimentar saudável diferiu segundo o hábito de realizar refeições sem telas (p-valor<0,001) e o hábito de comer à mesa (p-valor<0,001). O hábito de comer em companhia não se relacionou ao escore de consumo alimentar saudável (p-valor 0,199) (Tabela 4). 

**Tabela 4:** Práticas de comensalidade segundo marcadores de consumo alimentar não saudável de adultos usuários da Atenção Primária à Saúde. Goiânia, 2022-2023 (n=783)

Variáveis	Marcadores não saudáveis												Escore de consumo alimentar saudável (n=783)	
	Hambúrguer e/ou embutidos (n=783)			Bebidas adoçadas (n=783)			Macarrão instantâneo, salgadinhos de pacote ou bolacha salgada (n=783)			Bolacha recheada, doces ou guloseimas (n=783)				
	Sim n (%)	Não n (%)	p-valor	Sim n (%)	Não n (%)	p-valor	Sim n (%)	Não n (%)	p-valor	Sim n (%)	Não n (%)	p-valor	Mediana (IIQ)	p-valor
**Amostra total^f^**	152 (19,4)	631 (80,6)	-	408 (52,1)	375 (47,9)	-	143 (18,3)	640 (81,7)	-	224 (28,6)	559 (71,4)	-	5 (4-6)	-
**Hábito de realizar as refeições sem o uso das telas**			0,001^c^			<0,001^c^			0,056^c^			0,001^c^		<0,001^b^
Sim	110 (72,4)	366 (58,0)		272 (66,7)	204 (54,4)		97 (67,8)	379 (59,2)		156 (69,6)	320 (57,2)		5 (5-6)	
Não	42 (27,6)	265 (42,0)		136 (33,3)	171(45,6)		46 (32,2)	261 (40,8)		68 (30,4)	239 (42,8)		5 (4-6)	
**Hábito de consumir alimentos à mesa**			0,591^c^			0,010^c^			0,922^c^			0,722^c^		<0,001^b^
Sim	98 (64,5)	392 (62,1)		238 (58,3)	252 (67,2)		90 (62,9)	400 (62,5)		138 (61,6)	352 (63,0)		5 (4-6)	
Não	54 (35,5)	239 (37,9)		170 (41,7)	123 (32,8)		53 (37,1)	240 (37,5)		86 (38,4)	207 (37,0)		5 (4-6)	
**Hábito de consumir alimentos com companhia**			0,923^c^			0,701^c^			0,320^c^			0,264^c^		0,199^b^
Sim	117 (77,0)	488 (77,3)		313 (76,7)	292 (77,9)		115 (80,4)	490 (76,6)		179 (79,9)	426 (76,2)		5 (4-6)	
Não	35 (23,0)	143 (22,7)		95 (23,3)	83 (22,1)		28 (19,6)	150 (23,4)		45 (20,1)	133 (23,8)		5 (4-6)	
**Escore de práticas de comensalidade recomendadas** (n=783) Mediana (IIQ)	2 (1-2)	2 (1-3)	0,149^b^	2 (1-2)	2 (1-3)	0,001^b^	2 (1-2)	2 (1-3)	0,526^b^	2 (1-2)	2 (1-3)	0,163^b^	-	-

^a^Valores apresentados em mediana (variação interquartil); ^b^Teste de U de Mann-Whitney; ^c^Teste qui-quadrado de Pearson; ^d^Teste exato de Fisher; ^e^Teste Kruskal-Wallis; ^f^Algumas variáveis apresentam n<783 devido aos dados faltantes.

O escore de práticas de comensalidade recomendadas foi diferente segundo idade (p-valor<0,001), raça/cor da pele (p-valor 0,003), uso de tabaco (p-valor<0,001) e nível de atividade física (p-valor<0,001). Consumir alimentos sem telas foi maior entre participantes mais velhos (p-valor<0,001), que não utilizavam álcool (p-valor 0,035) e sedentários (p-valor 0,005). Comer à mesa foi mais frequente entre os participantes mais velhos (p-valor 0,006), de raça/cor da pele parda (p-valor<0,001), com renda entre 2-3 salários mínimos (p-valor 0,001), que não usavam tabaco (p-valor<0,001) e sedentários (p-valor 0,000). Comer em companhia foi maior entre aqueles que não usavam tabaco (p-valor 0,001) (Tabela suplementar 1).

Houve correlação positiva entre os escores de práticas de comensalidade recomendadas e consumo alimentar saudável (ρ=0,208, p-valor<0,001) (dados não apresentados em tabelas).

Na análise ajustada para a amostra total, a cada prática de comensalidade recomendada a mais, houve aumento do escore de consumo alimentar saudável em 0,33 (IC95% 0,11; 0,56, p-valor 0,004, t=2,91) no percentil 10; 0,30 (IC95% 0,14; 0,47, p-valor<0,001, t=3,65) no percentil 25; 0,17 (IC95% 0,06; 0,29, p-valor=0,004, t=2,91) no percentil 50; e 0,15 (IC95% 0,02; 0,28, p-valor 0,019, t=2,34) no percentil 75, contudo não esteve associado (IC95% 0,13; -0,08; 0,34, p-valor 0,222, t=1,22) ao percentil 90 da distribuição do escore de consumo alimentar saudável. A análise ajustada estratificada por sexo mostrou que, para o sexo feminino, a cada prática de comensalidade recomendada a mais, houve aumento do escore de consumo alimentar saudável em 0,42 (IC95% 0,18; 0,66, p-valor 0,001, t=3,46) no percentil 10; 0,31 (IC95% 0,13; 0,49, p-valor 0,001, t=3,44) no percentil 25; 0,17 (IC95% 0,04; 0,29, p-valor 0,008, t=2,66) no percentil 50; e 0,16 (IC95% 0,02; 0,31, p-valor 0,027, t=2,21) no percentil 75, mas não esteve associado (0,17; IC95% -0,04; 0,38, p-valor 0,123, t=1,55) ao percentil 90 da distribuição do escore de consumo alimentar saudável. Não foram encontradas associações para o sexo masculino (Tabela 5). 

**Tabela 5:** Coeficiente com intervalo de confiança de 95% (IC95%) da associação entre práticas de comensalidade e os percentis da distribuição dos marcadores de consumo alimentar do dia anterior de adultos usuários da Atenção Primária à Saúde. Goiânia, 2022-2023

Modelo	Percentil 10		Percentil 25		Percentil 50		Percentil 75		Percentil 90	
	Coeficiente (IC95%)	p-valor	Coeficiente (IC95%)	p-valor	Coeficiente (IC95%)	p-valor	Coeficiente (IC95%)	p-valor	Coeficiente (IC95%)	p-valor
Total										
Modelo bruto^a^ (n=783)	0 (-0,23; 0,23)	1,000	0,50 (0,29; 0,71)	<0,001	0 (-0,14; 0,14)	1,000	0 (-0,09; 0,09)	1,000	0 (-0,12; 0,12)	1,000
Modelo ajustado^b^ (n=710)	0,33 (0,11; 0,56)	0,004	0,30 (0,14; 0,47)	<0,001	0,17 (0,06; 0,29)	0,004	0,15 (0,02; 0,28)	0,019	0,13 (-0,08; 0,34)	0,222
**Sexo feminino**										
Modelo bruto^a^ (n=639)	0,5 (0,26; 0,74)	<0,001	0,50 (0,28; 0,72)	<0,001	0 (-0,18; 0,18)	1,000	0 (-0,09; 0,09)	1,000	0 (-0,12; 0,12)	1,00
Modelo ajustado^b^ (n-576)	0,42 (0,18; 0,66)	0,001	0,31 (0,13; 0,49)	0,001	0,17 (0,04; 0,29)	0,008	0,16 (0,02; 0,31)	0,027	0,17 (-0,04; 0,38)	0,123
**Sexo masculino**										
Modelo bruto^a^ (n=144)	0 (-0,54; 0,54)	1,000	0,33 (-0,06; 0,73)	0,101	0,33 (-0,03; 0,70)	0,075	0 (-0,29; 0,29)	1,000	0,33 (-0,32-0,99)	0,318
Modelo ajustado^b^ (n=134)	-0,02 (-0,54; 0,50)	0,931	0,42 (-0,06; 0,90)	0,089	0,14 (-0,25; 0,52)	0,481	0,04 (-0,37; 0,45)	0,834	-0,15 (-0,45; 0,14)	0,311

^a^Análise de regressão quantílica adotando como desfecho o escore de alimentação saudável (0-7 pontos) e como exposição o escore de práticas de comensalidade recomendadas (0-3 pontos) em modelos brutos; ^b^Análise de regressão quantílica adotando como desfecho o escore de alimentação saudável (0-7 pontos) e como exposição o escore de práticas de comensalidade recomendadas (0-3 pontos) em modelos ajustados pela idade, sexo (apenas para a amostra total), escolaridade, índice de massa corporal, nível de atividade física percebida, uso de álcool e uso de tabaco.

## Discussão 

Este estudo identificou que a adesão às práticas de comensalidade recomendadas pelo Guia, como comer à mesa, em companhia e sem telas, foi associada ao consumo alimentar mais saudável em mulheres. As análises ajustadas mostraram que, a cada prática adicional, o escore de consumo saudável aumentava, exceto entre aquelas com escores já elevados. Essa associação não foi observada em homens.

Neste estudo, foi estabelecido um escore para mensurar as práticas de comensalidade, cuja mediana foi moderada. Comer em companhia foi a prática mais frequente, seguido por comer à mesa, enquanto a menor prevalência foi a de refeições sem telas. A mediana do escore de consumo alimentar saudável foi elevada, devido à maior frequência de consumo de alimentos in natura, minimamente processados, e preparações culinárias como alimentos do grupo que incluem frutas frescas, hortaliças e feijões, em conformidade com a regra de ouro do Guia (3).

Apesar de dados nacionais revelarem que o consumo de feijão sofreu redução ao longo dos anos (5), esse foi o marcador saudável mais prevalente neste estudo. O consumo de feijão é um hábito importante para a qualidade da dieta, pois favorece o consumo de alimentos saudáveis como arroz, salada e carne (13). Porém, observou-se que mais da metade da amostra consumiu bebidas adoçadas, como refrigerantes, sucos de frutas artificiais e de frutas adoçadas. O consumo de suco de frutas é comum durante a refeição (14). No entanto, o Guia recomenda o consumo de frutas inteiras ao invés de sucos, visto que estes apresentam menos fibras e menor saciedade (3). Apesar da redução acumulada de 43,8% de 2007 a 2019 quanto ao consumo de refrigerantes/sucos artificiais, este permanece elevado (15), como observado. 

Os indivíduos com mais de 36 anos, do sexo feminino, muito ativos, que não usam tabaco ou álcool, que se alimentam sem o uso de telas e que consomem alimentos à mesa apresentaram maiores escores de alimentação saudável. Observou-se, em adultos brasileiros, tendência de melhora nas práticas alimentares com o aumento da idade em ambos os sexos (16). Na Pesquisa Nacional de Saúde (2019), segundo sexo, faixa etária e escolaridade, as mulheres, os indivíduos mais jovens e aqueles com maior escolaridade apresentaram maior proporção de consumo recomendado de frutas, hortaliças e peixes, e menor proporção de consumo de refrigerantes, sucos artificiais e doces (17). O sexo masculino esteve associado ao maior consumo de ultraprocessados. Esses fatores devem ser considerados em políticas públicas que promovam equidade alimentar (18).

Análises de regressão quantílica ajustadas pelas variáveis sociodemográficas, pelo estilo de vida e pelo estado nutricional foram empregadas para investigar a associação entre escore de práticas de comensalidade recomendadas e o escore de consumo alimentar saudável em diferentes pontos da distribuição. Essa abordagem se mostrou apropriada diante da assimetria da distribuição do escore de consumo alimentar saudável. 

Nos modelos ajustados para a amostra total, observou-se que, a cada prática de comensalidade recomendada adicional, houve aumento significativo do escore de consumo alimentar saudável nos percentis 10, 25, 50 e 75 da distribuição, enquanto nenhuma associação foi detectada no percentil 90. Esses resultados mostraram que os indivíduos nos menores percentis de distribuição foram os que mais se beneficiaram com a adoção das práticas de comensalidade, possivelmente devido à maior margem para melhorias no seu consumo alimentar. Para indivíduos com alimentação mais saudável, a possibilidade de mudanças adicionais na dieta foi mais restrita (19). Outros fatores podem proteger esses indivíduos, como educação alimentar e nutricional, autocontrole alimentar, preocupação com a saúde, disponibilidade e acesso a alimentos saudáveis, estilos de vida saudáveis, saúde mental e emocional (20-22), comer com atenção, regularidade e dedicar mais tempo à alimentação (3).

A análise estratificada por sexo revelou resultados distintos. Entre as mulheres, houve associação positiva entre o escore das práticas de comensalidade e o escore de alimentação saudável em vários percentis, exceto no percentil 90 da distribuição, como observado no modelo para a amostra total. Nenhuma associação significativa foi observada entre os homens, mesmo após o ajuste por potenciais fatores de confusão. A falta de associação entre os homens pode estar relacionada tanto ao tamanho reduzido da amostra masculina quanto à menor adesão a práticas de comensalidade ou devido a outros fatores não ajustados. 

Diferenças entre os sexos podem incluir aspectos comportamentais, sociais e culturais. As mulheres têm maior envolvimento com a comensalidade e atividades que envolvem a alimentação, o que favorece maiores habilidades culinárias e influencia o consumo alimentar mais saudável. O consumo alimentar masculino pode apresentar maior influência de fatores como praticidade, saciedade e hábitos automatizados, o que pode atenuar ou neutralizar o impacto da comensalidade nesse grupo (23). Problematizar esse padrão cultural pode incentivar a participação masculina nessas práticas e favorecer o consumo alimentar saudável entre os homens (24). 

Quando expostas a orientações sobre saúde, mulheres tendem a adotar comportamentos mais saudáveis (25). Comer assistindo televisão é uma prática que se contrapõe ao ato de compartilhar refeições à mesa e em companhia. Comer em companhia melhora a qualidade da dieta, reforça laços sociais e culturais, promove estilo de vida mais saudável (26,27) e se relaciona com o menor risco de obesidade (28). Em uma visão bioarqueológica, compartilhar alimentos e participar de refeições coletivas constituem atos que conectam o ser humano como um organismo biológico à sua natureza social (29), além da maior sensação de prazer proporcionada pela alimentação (30). Aderir às práticas de comensalidade recomendadas pelo Guia Alimentar para População Brasileira se associou à melhor qualidade da dieta (7). 

Os resultados deste estudo reforçaram a relevância da promoção de práticas de comensalidade pelas equipes na Atenção Primária à Saúde, como estratégia para melhorar a qualidade da alimentação e aproximá-la das diretrizes do Guia. Assim, destacaram-se a assertividade das recomendações multidimensionais do Guia e a publicação dos protocolos (31) para apoiar os profissionais de saúde. 

Algumas limitações devem ser pontuadas. Como foram incluídos no estudo apenas usuários adultos da Atenção Primária à Saúde de Goiânia, os resultados não podem ser extrapolados para outras faixas etárias e regiões do país, considerando a ampla diversidade cultural e alimentar. O nível de atividade física pode não refletir com precisão sua intensidade, por se basear na autopercepção dos participantes. Outra limitação foi a utilização de medidas de peso e estatura autorreferidas para parte da amostra, porém medidas aferidas apresentam alta concordância para medidas autorreferidas (32). Neste estudo, apresentou-se a variável estado nutricional categorizada, o que contribuiu para minimizar possíveis discrepâncias, uma vez que pequenas diferenças absolutas nas medidas tendem a não alterar a classificação dos indivíduos em categorias de índice de massa corporal (33). Apesar de a análise estratificada por sexo ter sido adotada para reduzir vieses, a perda diferenciada de homens pode ter comprometido a representatividade dos achados. Essa diferença pode estar relacionada ao menor engajamento masculino em estudos de saúde (34). 

Os pontos fortes deste trabalho incluíram a realização do estudo-piloto, a coleta de dados em todos os distritos sanitários de Goiânia e o uso do Formulário de Marcadores do Consumo Alimentar do Sistema de Vigilância Alimentar e Nutricional, adotado em nível nacional. A estrutura interna desse instrumento se mostrou estável quanto às evidências de invariância de mensuração entre macrorregiões brasileiras, às fases do curso da vida e ao longo do tempo (35). Além disso, são escassos na literatura estudos que avaliam práticas alimentares (7,16), especialmente de comensalidade. 

Conclui-se que a adesão às práticas de comensalidade recomendadas pelo Guia Alimentar para População Brasileira, como comer à mesa, em companhia e sem telas no momento das refeições, estão associadas ao consumo alimentar mais saudável entre usuárias adultas da Atenção Primária à Saúde de Goiânia. As associações não foram observadas em homens. A inclusão dessas recomendações em ações de promoção da saúde na Atenção Primária à Saúde pode contribuir para a melhor qualidade da alimentação da população brasileira. 

## MATERIAL SUPLEMENTAR

Tabela Suplementar 1
